# Piloting the feasibility of a population-based joint TB-HIV survey in KwaZulu-Natal Province, South Africa, 2019

**DOI:** 10.1371/journal.pgph.0005804

**Published:** 2026-01-28

**Authors:** Pelagia Murangandi, Olanrewaju Oladimeji, Nompumelelo Zungu, Lehlogonolo Makola, Edmore Marinda, Leickness Simbayi, Khangelani Zuma, Shandir Ramlagan, Sean Jooste, Goitseone Maseko, Inbarani Naidoo, Chijioke Nwosu, Stephen McCracken, Sizulu Moyo, Wolfgang Hladik

**Affiliations:** 1 Division of Global HIV & TB, Centers for Disease Control and Prevention, Pretoria, South Africa; 2 Department of Health Sciences, Faculty of Health Science, Durban University of Technology, Durban, South Africa; 3 Department of Public Health, School of Health Care Sciences, Sefako Makgatho Health Sciences University, Pretoria, South Africa; 4 Public Health, Societies and Belonging & Impact Centre, Human Sciences Research Council, Pretoria, South Africa; 5 School of Nursing and Public Health, University of KwaZulu-Natal, Durban, South Africa; 6 University of Johannesburg, SAMRC/UJ Pan African Centre for Epidemics Research (PACER), Johannesburg, South Africa; 7 Department of Psychiatry & Mental Health, University of Cape Town, Cape Town, South Africa; 8 School of Public Health, Biostatistics and Epidemiology Division, University of the Witwatersrand, Johannesburg, South Africa,; 9 Department of Economics and Finance, University of the Free State, Bloemfontein, South Africa; 10 Division of Global HIV & TB, Centers for Disease Control and Prevention, Atlanta, Georgia, United States of America; 11 School of Public Health and Family Medicine, University of Cape Town, Cape Town, South Africa; University of Sydney, AUSTRALIA

## Abstract

South Africa bears a high burden of infectious diseases, including intersecting epidemics of human immunodeficiency virus (HIV) and tuberculosis (TB). Among people living with HIV, TB is the predominant cause of death. Since 2002, South Africa has conducted six national population-based HIV surveys, and its first national TB prevalence survey between 2017 and 2019. Given the epidemiologic overlap of these conditions and dwindling resources, a joint national TB and HIV survey could be advantageous. We piloted a joint survey design in August–September 2019 to assess the feasibility of simultaneously collecting HIV and TB data. The pilot survey utilized the same sampling frame as the 2017–2019 national TB prevalence survey, based on small area layers as building blocks for clusters. Two clusters in KwaZulu-Natal (one urban and one rural) were selected. People of all ages were eligible to participate. Household questionnaires were administered to consenting household heads, followed by invitations to the cluster survey hub, where age-appropriate individual questionnaires were administered. Whole blood samples were tested for HIV, viral load, HIV drug resistance and HIV recent infection status. TB metrics included symptom and chest x-ray screening with sputum testing for those screening positive. Those ≥18 years received other health measurements (weight, height) and screening tests (random blood glucose, cholesterol). The survey successfully combined the collection of both HIV and TB relevant data. Overall, Household-level uptake was 78.6% (363/462), while individual-level uptake at the hub was 48.1% (616/1,280), with lower participation in the urban cluster. Uptake of additional health measurements exceeded 87%. The pilot study demonstrated that combining TB and HIV surveys is possible, but fewer people participated compared to the individual national HIV and TB surveys. Further operational research could explore how to optimize survey design, accommodate differing data requirements, and improve participation in future joint surveys.

## Introduction

South Africa has a high infectious disease burden with overlapping human immunodeficiency virus (HIV) and tuberculosis (TB) epidemics [1]. People living with HIV (PLHIV) are at greater risk of developing TB disease, and HIV is also frequently associated with TB [2,3]. In addition, TB is a leading cause of death among PLHV [4] in sub-Saharan Africa. With an estimated 7.6 million PLHIV in South Africa in 2019 [5] and 58% of people with TB coinfected with HIV [6], an integrated approach is important in controlling and monitoring these two epidemics.

South Africa has conducted multiple population-based national HIV prevalence, and behaviour (SABSSM) surveys since 2002 [7–11]. SABSSM surveys sample people of all ages in household settings and their interviews assess service uptake, risk behaviours and attitudes towards HIV. The 2017 and 2022 surveys also included questions on TB knowledge [11,12]. Dry blood spot (DBS) samples are collected for biomarker testing, including HIV serology, antiretroviral (ARV) drugs, recency of HIV infection, viral load (VL), and HIV drug resistance.

South Africa conducted its first national TB prevalence survey (SATPS) between 2017–2019. The survey targeted people aged 15 years and older and included symptom and chest radiography screening for TB. For those that screened positive, sputum samples were collected for *Mycobacterium tuberculosis (M.tb) testing and* optional DBS samples for HIV serological testing [13]. As in the SATPS, other national TB prevalence surveys in high TB and HIV burden settings have also included HIV testing in a subset of participants [14,15], but no survey included HIV and related tests for all survey participants. Likewise, to date, no national HIV population survey in South Africa, has also included TB screening and testing.

With TB being the most important coinfection among PLHIV, conducting a joint national TB-HIV survey could provide better insight into the double burden of these two diseases at the population level. Although the design differs for HIV and TB surveys – including variations in sample size, sampling methods, on and off-site testing approaches, sample collection procedures, storage, transport logistics and handling of participants who test positive — there is potential to conduct joint surveys as both typically use a household sampling frame with possible efficiencies in cost and effort [16,17].

A successful joint survey would inform both disease domains and ideally allow for the examination of TB and HIV comorbidity. Furthermore, TB prevalence surveys often have a per-participant cost of $100 or less [16]. SABSSM uses a similar sampling design as population-based HIV Impact Assessment (PHIA) – the current standard for national HIV household surveys [17] though with a less costly approach for biomarker collection. A joint survey may lead to cost savings compared to separate TB and HIV surveys.

In 2019 we conducted a small-scale pilot survey to gain experience and insight into the operational feasibility of a combined TB-HIV household survey and to estimate costs of a hypothetical scaled-up national joint survey. This paper reports on the feasibility of a combined TB-HIV survey.

## Methods

### Overall survey design and setting

While South Africa’s national HIV survey procedures are mostly implemented at the household level, TB surveys typically administer their main survey procedures at a hub within or near the selected clusters. For this joint survey pilot, we adopted the TB hub system and integrated the HIV survey procedures within it. The pilot joint TB-HIV survey used the same sampling frame as the SATPS, which was based on small area layers (SALs) as building blocks for clusters [18]. Two clusters in KwaZulu-Natal were selected – one urban and one rural. Table 1 shows the main characteristics of the HIV, the TB and the TB-HIV pilot survey.

**Table 1:** Key characteristics of the HIV survey (SABSSM), TB prevalence survey (SATPS) and TB-HIV Pilot Survey, South Africa, 2019.

	SABSSM (HIV)	SATPS (TB)	TB-HIV pilot
**Age**	All ages	15 + years	All ages
**Number of clusters**	1 000	110	2
**Sample size per cluster**	<50	~500	>500
**Residency definition (number of nights in household)**	1 night (night before survey)	10 nights (of the preceding 2 weeks)	5 nights (of the preceding 2 weeks)
**Field Team staff size**	<10	>10	17*
**Compensation**	None	Some	Some
**Return of results**	Yes	Yes	Yes
**Sample collection/ type**	Capillary blood, DBS	CXR, sputum, DBS	CXR, sputum, venous/capillary blood, DBS
**Biomarker domains**	HIV	TB	HIV, TB, NCDs

SABSSM: SATPS: South Africa TB prevalence survey, DBS: dried blood spots, CXR: chest X-ray, NCD: non-communicable diseases, * Excludes 8 local volunteers in each cluster.

#### Sample size, location, and sampling period.

We did not intend this joint survey to be powered for any particular outcome, such as HIV or TB prevalence; rather, our target sample sizes, up to 500 adults and their accompanying children in each cluster were meant to be large enough to meaningfully assess the feasibility, logistics and economic cost of implementing a joint HIV/TB survey. The joint survey was conducted in KwaZulu-Natal from August 26 to September 14, 2019. All survey procedures were implemented within nine consecutive days in each cluster; survey activities included a household census (Days 1–3), set up of survey hub (Day 3), hub data collection activities (Days 4–8), and wrap up and closure of the hub (Day 9). Hub activities were conducted during the day and early evenings and were scheduled to overlap with weekends to enable participation from working household members within each cluster.

#### Survey staff and training.

Fieldwork staff (n = 17) were recruited from personnel who had previously worked on the HIV or TB surveys or other HIV or TB studies. The team comprised of one team leader, one medical officer, one receptionist, five interviewers, two phlebotomists, three nurses, one radiographer, one field information technology (IT) technician, one data checker, and one driver. In addition, community volunteers were recruited through leadership structures in each cluster. These volunteers were members of the field survey team serving a liaison function with local authorities and their community during the survey. The training lasted nine days for fieldwork staff, and a field competency test was conducted. Community volunteers received one day of training in preparation for survey activities.

#### Cluster locations.

Two clusters, one urban and one rural, were selected. Both selected clusters were adjacent to clusters previously sampled in the recently completed SATPS [18], but had not been utilized in either the SATPS or the national HIV survey (2017) [11]. A single survey hub was established in a convenient location in each cluster, i.e., a church (in the urban cluster) and a community centre (in the rural cluster).

#### Cluster level procedures.

The survey team conducted pre-survey engagements with community stakeholders and shared information about the forthcoming survey. This included survey team members holding a community meeting in the rural cluster to explain the aim and objectives of the pilot survey and survey processes to community members and leaders as part of community mobilization. Thereafter a situation assessment to determine the best location of the survey hub was conducted. A household census of people residing in the households was then undertaken. During this phase, survey field staff, along with community volunteers, encouraged community members in households to participate in the survey. Every household and all eligible people in the two clusters were invited to participate by attending the hub for survey procedures. The invitations considered people’s availability and specified the date and time when they could visit the hub. This approach aimed to help the survey team manage people efficiently at the hub, ensuring shorter waiting times for survey procedures.

Appointments helped with planning hub activities including follow-ups of non-attendees via direct text messages and attending to appointments to transport participants to and from the hub. Mobilisation of invited participants to the hub continued throughout the survey period in each cluster. Up to three phone calls and three home visits were done and these could be done simultaneously, e.g., making a follow up telephone call, and visiting the home on the same day. In addition, public announcements were made in the rural cluster.

#### Household procedures.

A household questionnaire (S1 Text) was administered to the household head; this questionnaire listed all household members and determined their eligibility for individual survey enrolment. The eligibility criteria for the pilot joint survey as well as the national HIV and TB surveys are shown in Table 1. All eligible household residents were assigned a survey ID number and were invited to present at the survey hub for enrolment.

#### Survey hub set up.

The hub had a multi station arrangement, with the following designated stations, reception, a waiting area, group information, individual consent (written or verbal — assent and parental or guardian consent was required for those <18 years old) and interview, chest- x-ray (CXR), non-communicable diseases (NCD) measurements, blood collection and HIV rapid testing, medical officer (MO), sputum collection, and an exit station. Given that the hub structure was adopted from the TB survey, the set up for TB survey related processes, such as infection prevention control measures, closely resembled that of South Africa’s TB survey. Sputum collection was undertaken in mobile sputum collection booths that were positioned to ensure appropriate airflow. Confidentiality during the interviews was maintained through physical distance between interview stations and HIV testing was conducted in a mobile clinic/van parked at the hub.

#### Interview.

Separate individual questionnaires (S1 Text) were interviewer-administered to parents or guardians for children 11 years or younger, and directly to those aged 12–14 years, and 15 years and older. These questionnaires were available in English and isiZulu. Every 5^th^ participant 15 years and older arriving at the hub was selected for computer-assisted self-interviewing (CASI) and given a brief tutorial on its use. All interview data were collected and managed using the Research Electronic Data Capture (REDCap) system. The questionnaires included questions adapted from the respective national HIV and TB prevalence surveys. HIV status awareness was probed during the individual interview at the hub and during the HIV counselling and testing process.

#### CXR and biomarker sample collection.

Participants 15 years and older underwent CXR screening on-site provided through mobile digital CXR. This was followed by measurements for height, weight, and blood pressure. Blood samples were drawn by venepuncture, finger prick, or heel prick in infants less than two years old, for HIV rapid testing (according to the national testing algorithm with pre and post-test counselling), other HIV related laboratory testing, random blood glucose, and cholesterol testing in those 18 years and older. Participants then proceeded to the MO station where their CXR findings were assessed by the MO (who had been trained in CXR reading) in combination with the TB screening questions (i.e., cough (persistent cough for ≥2 weeks or more or cough of any duration if HIV positive), unexplained fever for ≥2 weeks, drenching night sweats and unexplained weight loss (more than 1.5 kg in a month)). Two sputum specimens were obtained one hour apart from participants identified as having presumptive TB based on TB screening questions and or CXR findings. The MO also discussed the BMI, blood pressure and random blood glucose and cholesterol test results. Participants with out-of-range results including HIV-positive participants not on ART were given a referral form to take to a healthcare facility for further investigation. On completion of these procedures, the participants exited the hub.

#### Sample handling and testing procedures.

Dried blood spot (DBS) samples were prepared from blood samples collected with the remaining samples processed into plasma onsite and aliquoted into vials for testing at the central laboratory. The DBS, plasma, and sputum samples were appropriately labelled. Plasma samples were stored between 2–8 °C in a fridge or in a cooler bag with gel ice packs on-site and during transport. Sputum samples were collected into screw-top sputum jars and stored in a refrigerator on-site (for a maximum of 48 hours) before transport to the microbiology laboratory. The first sputum sample was tested on Xpert MTB/RIF Ultra (Xpert MTB/RIF Ultra (Xpert Ultra) (Cepheid, Sunnyvale, USA) and the second underwent culture on the MGIT 960 (BACTEC MGIT 960 System (Becton-Dickinson, Sparks, MD, USA)). The plasma samples were tested for HIV antibodies on the Cobas HIV1/2 (screening) and the Genscreen HIV1/2 (confirmatory) assays, viral load using the Abbott platform (Abbott m2000 HIV Real-Time System, Abbott Molecular Inc., Des Plaines, Illinois, USA), the limiting antigen avidity enzyme immunoassay (LAg Avidity EIA) (Maxim Bio-medical, Rockville, MD, USA) with a cut-off optical density for HIV recency, and genotyped (Next-generation sequencing performed using an in-house assay and amplicons sequenced using MiSeq v2 (Illumina Inc., San Diego, USA)) for HIV drug resistance. All HIV positive samples were confirmed using the BioRad Geenius HIV1/2 assay (Bio-Rad Laboratories, Inc. Hercules, California, USA). DBS cards were used as backup samples for the above-mentioned HIV tests. For children under 18 months, we used Roche Cobas Amplicor Taqman HIV1 DNA PCR (Roche Diagnostics Nederland B.V) to confirm HIV status. Participants could access the results of their laboratory tests at the clinic in the cluster after 4 days for sputum Xpert results, 8 weeks for sputum culture results, 12 weeks for HIV VL, discrepant HIV rapid test and central laboratory results and infant HIV results, and 6 months for HIV drug resistance results. Upon diagnosis of MTB in a specimen, a copy of the laboratory results was provided to the survey project directors and the TB coordinator of the area where the cluster was located for her/him to trace the individual concerned and to facilitate initiation of TB treatment.

#### Qualitative interviews.

To understand the implementation process, a small-scale qualitative study comprising focus group discussions (FGDs) with various staff and volunteers as well as key informant interviews were conducted with a nurse in-charge in each of the clinics located in the cluster and field staff (including volunteers) in the rural cluster. Field staff diaries and field observations provided additional data points.

### Ethics approval

This activity was reviewed by CDC, deemed research, and was conducted consistent with applicable federal law and CDC policy (See 45 C.F.R. part 46.101(c); 21 C.F.R. part 56). In addition, this pilot joint survey was reviewed and approved by the Research Ethics Committee of the Human Sciences Research Council (HSRC). Fieldworkers received training in ethics and survey procedures. Modest in-kind reimbursements to the value of approximately $3.57 were provided to survey participants who were enrolled at the survey hub. The reimbursements were in the form of food vouchers, airtime vouchers, washing powder or soap bars for adults. For participating children, the reimbursement included toys, colouring books, and crayons to the same value of $3.57.

#### Inclusivity in global research.

Additional information regarding the ethical, cultural, and scientific considerations specific to inclusivity in global research is included in the Supporting Information (S2 Text)”

## Results

*Survey response rates*. An overall total of 462 households were approached, and 363 (78.6%) household heads were interviewed; 62.2% (161/269) in the urban cluster, and 99.5% (202/203) in the rural cluster. Almost all (1 280/1 290, 99.2%) eligible people initially accepted an invitation to attend the survey hub; but the realised proportion of eligible people presenting at the hub was 48.1% (616/1 280); 38.2% and 55.4% in the urban and rural cluster respectively (Table 2).

**Table 2:** Enrolment at the hub by sex, age and cluster, TB-HIV Pilot Survey, South Africa, 2019.

	Individuals who accepted invitations at the household n	Individuals who attended the hub and were enrolled n (%)
**Total**	**1 280**	**616 (48.1)**
**Sex**		
Male	576	228 (39.5)
Female	704	388 (55.1)
**Age group (years)**		
0–11	294	148 (50.3)
12–14	73	30 (41.1)
15+	913	438 (48.0)
**Cluster**		
Urban	542	207 (38.2)
Rural	738	409 (55.4)

Of the 438 enrolled survey participants who were 15 years and older, 120 (27.4%) screened positive for presumptive TB based on symptoms and/or chest X-ray and submitted sputum samples for testing (Table 3). Samples from three participants were positive on Xpert MTB/RIF Ultra and none were positive on culture. Of the 616 participants who attended the hubs, 423 (68.7%) accepted HIV rapid testing and 62.8% (387/616) provided venous blood for HIV testing with 15.2% (59/387) testing positive for HIV (Table 4).

**Table 3:** TB screening and samples collected for laboratory testing by cluster among participants 15 years and older, TB-HIV Pilot Survey, South Africa, 2019.

Variable	Total (N)	Urban cluster (n)	Rural cluster (n)
Participants screened for TB*	438	169	269
Screened positive for TB*	120	33	87
**Samples received for Xpert MTB/RIF Ultra assay**	120	33	87
**Samples received and processed for culture**	119	32	87

*TB screening included symptoms and chest x-ray screening.

**Table 4:** HIV testing, HIV results and viral load results, TB-HIV Pilot Survey, South Africa, 2019.

	Total n (%) n = 616	Urban cluster n (%) n = 207	Rural cluster n (%) n = 409
**HIV RT sample** ^**#**^	423 (68.7)	127 (61.4)	296 (72.4)
**Tested positive on HIV RT**	33 (7.8)	1 (0.8)	32 (10.8)
**Venous blood sample**	387 (62.8)	103 (49.8)	284 (69.4)
Tested positive in the laboratory**	59 (15.2)	2 (1.9)	57 (20.1)
**Viral load^**	59	2 (100)	57 (100)
**Samples ≥1,000 copies/ml^**	11 (18.6)	1 (50.0)	10 (17.5)
**Samples <1,000 copies/ml^**	48 (81.4)	1 (50.0)	47 (82.5)

# % of those enrolled, ** % among those tested, ^%among those who tested HIV positive.

Uptake of other health screening tests and measurements (as described in Methods) was 87.6% or higher for these tests (Table 5).

**Table 5:** Glucose, cholesterol, and BMI measurement among participants 18 years and older, TB-HIV Pilot Survey, South Africa, 2019, n = 415.

	Total n (%) n = 415	Urban cluster n (%) n = 161	Rural cluster n (%) n = 254
**Participants with random blood sugar reading**	366 (88.2)	143 (88.8)	223 (87.8)
**Participants with cholesterol reading**	364 (87.7)	141 (87.6)	223 (87.8)
**Participants with body mass index calculated**	381 (91.8)	154 (95.7)	227 (89.4)

The minimal dataset underlying this paper is included in the Supporting Information (S1 Dataset, S2 Dataset and S3 Text).

### Informal observations of survey implementation

In this manuscript we report on field observations and biomarker results, and not on the findings from the interviews. Our informal observations and structured discussions among staff and volunteers at the end of each workday contributed to a better understanding of this pilot’s success and challenges. The organisation of activities at the hub was intended to ensure time-efficiency. The survey was arranged in a way that after verification of invitation, informed consent for all procedures was the first activity after the group information session. The overall process was successfully executed, with bottlenecks emerging during the administration of informed consent and the CASI questionnaires. Issues related to CASI were primarily attributed to readability (the questionnaire had been designed as interviewer-administered) and participants’ confidence in using the electronic interface, leading to delays in data input. Connectivity issues further contributed to delays in the overall data entry process.

Completing some procedures at the hub; specifically rapid HIV testing with pre- and post-test counselling was lengthy and led to long waiting times. In the rural cluster participants disregarded their appointment times and many arrived at the hub early on the first day of hub activities. This overwhelmed the hub capacity and staff, leading to even longer waiting times and disruptions in the hub flow and organization.

The laboratory, located directly within the survey hub, while very helpful for the central laboratory, was labour intensive for the field with the staff and materials that were allocated. Blood sample processing on site prolonged hub operating hours because the field laboratory’s capacity was limited. Tests for blood sugar, cholesterol, and body mass index calculations were perceived as attractive to participants and contributed to survey enrolment. The late hub hours were also exacerbated because some participants could only attend the hub at the end of the day after their work schedules. However, processing of blood samples and courier of plasma, DBS, and sputum samples to testing laboratories adhered to protocol specified standards and overall was deemed successful.

The time allocated for household census and hub activities was not adequate to reach the target survey sample size due to various dynamics in the field. For example, the terrain in the rural cluster was challenging to access even by vehicle, thus prolonging the household census phase, and in both clusters some people worked outside the cluster, leaving their homes early and returning late in the evening – which made it difficult for them to participate in the survey. TB surveys usually include a flexible hub schedule that can extend the time in each cluster to increase participation. This option was not implemented in this pilot.

Although all members of the field team passed the competency testing conducted after training, it was evident that more intensive training would have been beneficial because those with more HIV survey experience gravitated towards HIV surveillance protocols and vice versa for those more experienced in TB field data collection, which occasionally led to slightly differing practices with hub activity flow and staff roles.

## Discussion

We successfully piloted a joint TB-HIV survey, using a hub-based survey approach typical for TB prevalence surveys with added HIV-specific interview and biomarker data collection. Overall, survey uptake was lower compared to South Africa’s 2018 national TB prevalence and South Africa’s 2017 and 2022 national HIV survey (SABSSM V and VI) [11,18,19]. Uptake of HIV testing (68.7%) was comparable to testing uptake among those who attended our survey pilot’s hubs to testing uptake among those who agreed to be interviewed and provided a blood sample (23 923/36 609, 61.1% and 47 966/76 134, 62.7%) in the 2017 and 2022 national SABSSM surveys [11,19]. However, only 48.1% of those accepting invitations to the hub were successfully enrolled in this pilot, whereas over 90% of eligible participants in SABSSM VI participated in the individual interview [19]. Unlike the current pilot where hubs were used to incorporate TB testing, SABSSM VI completed all survey procedures in households. It is also important to note that, a proportion of participants provided venous blood samples but declined rapid HIV testing contributing to the discrepancy observed between HIV-positive results obtained through hub-based rapid testing and those identified through central laboratory assays.

Among those screening positive for TB, our survey successfully collected and tested sputum samples for all, whereas in the national TB prevalence survey, samples could not be obtained in approximately 20% of screen-positive participants [18]. Differences in survey uptake and testing proportions can be attributed to several reasons. Firstly, the low overall survey uptake of 48% could be because some people were not keen to travel to the hub despite transport being provided as needed. Secondly, others did not want to spend time at the hub. Despite scheduling of hub appointments on different days to address congestion, in the rural cluster participants disregarded this and many presented to the hub on the first day of hub activities. This overwhelmed the hub staff capacity, resulting in prolonged stays at the hub. Thus, messages about long waiting times could have subsequently discouraged some from participating. The other contributing factor was people working outside the clusters, affecting their availability during the week, a challenge also encountered in other hub-based/ household-based surveys in South Africa. Additionally, survey activities may have conflicted with other community activities including funerals on the weekend in the rural cluster [18]. The first few days of the hub activities in the urban cluster were also affected by rain. Although participants were offered transportation, the schedules may have been unsuitable for some.

The survey combined TB and HIV components, including the collection of venous blood samples for HIV testing – an approach that has not yet been implemented in the series of national HIV surveys previously undertaken in South Africa. Although the field laboratory sample processing was labour intensive, it enabled use of plasma samples with superior range for VL detection when compared to DBS that has a higher lower limit of detection (LDL). Use of plasma samples for VL detection aligns more closely with current clinical guidelines where detection of VL as low as 50 copies/ml is significant to guide clinical decisions [20], is consistent with other PHIAs [21] and aligns with the VL cut off for U = U (Undetectable = Untransmittable) [22].

Increase in hub laboratory capacity could have improved its efficiency. Sputum sample collection was high; all participants eligible for sputum examination provided a sample. It is however notable that even with this small sample size one participant did not have a sample for culture and there was sample contamination (4.2%), although within the acceptable range [23]. This indicates the significance of sample handling and laboratory procedures both in the field and in the laboratory. In South Africa’s 2018 national TB prevalence survey approximately 20% of participants who screened positive did not have a valid culture result and the contamination rate was 5.4%, which was within an acceptable range [13,18].

There was high uptake of the other health assessments and tests done at the hub indicating interest in health conditions beyond HIV and TB. NCDs are a rising health burden on South Africa [24]. Including other screening tests in HIV and TB surveys together with clear referral pathways could increase participation, provide community level screening as well as reach those who do not normally interact with the healthcare system. Overall, the biomarker data obtained in our pilot was of good quality, with only a few missing variables. With TB as HIV’s most important co-infection and leading cause of death among PLHIV, a survey design that benefits from the lower (per participant) TB survey costs while integrating HIV metrics can provide helpful information for future surveys.

There are, however, formidable challenges to a joint survey design. As mentioned above, participation was poor, especially among adolescents and males, with less than 50% survey uptake overall. With more participants surveyed, increased survey team proficiency in administering survey processes may lead to higher survey uptake. TB prevalence surveys generally have lower participation rates compared to HIV or other surveys where data collection occurs within the household [25,26] – possibly due to the active effort required from the participant to reach the survey hub. While a hub design offers more efficient set-up with higher sample sizes per staff time unit, additional effort could attract a high enough proportion of potential survey participants to the cluster survey hub. In this survey pilot, transport to the hub was provided at specified times and participants were compensated for their time. A higher reimbursement with addition of small non-monetary prizes to randomly selected participants [27] may have further increased survey uptake, although there is need to maintain the voluntary nature of participation. Other benefits likely also attracted certain participants, such as random blood glucose and cholesterol testing, blood pressure and anthropometric measurements. These NCD measures though mostly appeal to an older demographic. Additional benefits specifically appealing to younger people including males both of which are traditionally more reluctant to participate in surveys could be beneficial.

Our joint survey design significantly lengthened the individual questionnaire as TB survey interviews are generally much shorter than those used in HIV surveys, e.g., in SABSSM or PHIA. A re-evaluation of joint survey interview metrics should yield a shorter interview length (45 minutes or less) by trimming to only include the (HIV) metrics that are essential for weighted data analysis, HIV estimation, and programming. Similarly, while the piloted self-administered data collection mode (CASI) was a challenge to complete for some participants, particularly the older ones, this was partly due to preparatory limitations that could be easily remedied. CASI has been used in African household settings before and a carefully prepared CASI instrument holds the promise of time savings for survey staff while facilitating more honest responses to sensitive questions [28].

To address prolonged hub times, an alternative survey design could shift the household and individual interview, the spot sputum sample, screening for NCDs and the HIV rapid testing to the household level, with participants only attending the hub for CXR, venous blood draws and for review by the MO. Further, computer-aided detection (CAD) has been shown to speed up fieldwork and reduce the need for molecular testing which may help making the survey more efficient and increase acceptability among participants [29]. To address hub laboratory limitations, we also suggest making use of a satellite laboratory that could operate independent of the screening and testing hub, as done in PHIAs [21,30].

Other improvements to a future joint survey may also include extending training time for field staff and improving flexibility on hub operation time and number of days spent in a cluster to address variations between clusters. Due to the influx of people into the hub despite scheduled appointments, supplementing the staff component in the first few days of hub activities could assist in reducing waiting times for certain sections of the hub activities.

The supplementary file (S4 Text) describes a possible sampling design for a national joint TB-HIV survey. TB surveys typically include far fewer survey clusters (often 50–100) [31] than HIV surveys (often close to 500 for a truly joint survey design and to satisfy both surveys’ primary objectives, the number of clusters would need to be closer to that required for subnational HIV viral load suppression estimation, while still collecting data via a central cluster location. That implies more effort than for a typical TB prevalence design, though perhaps less effort than for a typical HIV household survey design. Sample size considerations too are important as TB surveys, despite the small number of clusters typically require larger total sample sizes than HIV surveys. This may be addressed by collecting joint TB-HIV metrics only for as many as needed for HIV estimation, and to restrict the remaining sample to TB only metrics, or to TB plus (fingerstick-based) HIV rapid testing only. However, this may limit such a joint survey’s capacity to fully inform both (co-)morbidities.

The 1^st^ generation PHIA [17] also sampled children under 15 years of age, whereas the TB prevalence surveys focus on 15 + year-olds only. Meanwhile, 2^nd^ generation PHIA generally dropped paediatric sampling [32], so that this may no longer be a constraint for a joint survey design. However, in the South African HIV survey all ages are included. Hence, a joint survey design may drop paediatric sampling as well, or, alternatively, include metrics important to child health to increase participation.

The differing sample size requirements, sampling designs, and levels of data collection (hub vs. household) inherently impedes an efficient joint TB-HIV survey design. Nevertheless, the substantial investments necessary to survey each disease domain and the syndemic nature of these two diseases continue to call for a common survey design. Table 6 summarizes the key challenges discussed, their implications, and suggested approaches to addressing implementation challenges in future joint surveys.

**Table 6:** Summary of challenges and suggested approaches to address challenges in future implementations, TB-HIV Pilot Survey, South Africa, 2019.

Challenge identified	Description/ evidence	Implication	Potential solution or future consideration
Low participation rates	Overall survey uptake was **below 50%**, with particularly poor participation among **adolescents and males**. (Although, this is not surprising in South Africa especially among males).	Limits representativeness and feasibility of population-based estimates.	Strengthen community engagement, use flexible scheduling, and integrate youth- and male-focused mobilization strategies.
Complexity of joint survey design	Conducting a combined TB–HIV survey requires coordination across multiple teams, data systems, and diagnostic workflows.	Increases operational complexity and resource requirements.	Implement integrated training and streamline data collection and referral pathways.
Learning curve for survey teams	Proficiency improved as more participants were surveyed, suggesting initial inefficiencies.	Early clusters may have lower uptake or longer completion times.	Conduct extended pre-survey training and phased roll-out to build capacity before full implementation.

## Conclusion

We conducted a successful pilot of a joint TB-HIV survey, gathering valuable information to guide further refinement of this approach. The successful integration of these surveys can provide crucial insights into TB and HIV coinfection, allowing for a more efficient allocation of funds for TB and HIV programs, including surveillance.

## Supporting information

S1 Text Data collection tools. (DOCX)

S2 Text Checklist. (DOCX)

S3 Text Data dictionary. (DOCX)

S4 Text Supplementary information. (DOCX)

S1 Dataset Minimal household dataset. (DTA)

S2 Dataset Minimal individual dataset. (DTA)

## References

[1] World Health Organization. Global Tuberculosis Report 2022 [Internet]. 2022. Available from: https://www.who.int/teams/global-programme-on-tuberculosis-and-lung-health/tb-reports/global-tuberculosis-report-2022

[2] UNAIDS. Tuberculosis and HIV. Vol. 54, Joint United Nations Programme on HIV/AIDS. 2018.

[3] Sultana ZZ, Hoque FU, Beyene J, Akhlak-Ul-Islam M, Khan MHR, Ahmed S, et al. HIV infection and multidrug resistant tuberculosis: a systematic review and meta-analysis. BMC Infect Dis. 2021;21(1):51. doi: 10.1186/s12879-020-05749-2 33430786 PMC7802168

[4] UNAIDS. Fact Sheet — World Tuberculosis Day 2022 [Internet]. 2022 [cited 2023 Aug 31]. Available from: https://www.unaids.org/sites/default/files/media_asset/20220324_TB_FactSheet_en.pdf

[5] Johnson L, Meyer-Rath G, Dorrington R, Puren A, Seathlodi T, Zuma K. The Effect of HIV Programs in South Africa on National HIV Incidence Trends, 2000-2019. J Acquir Immune Defic Syndr. 2022;90(2):115–23. 35125471 10.1097/QAI.0000000000002927

[6] World Health Organization. Global Tuberculosis Report. 2020.

[7] Shisana O, Simbayi L. Nelson Mandela/HSRC Study of HIV/AIDS: South African National HIV Prevalence, Behavioural Risks and Mass Media Household Survey 2002. Cape Town: HSRC. 2002.

[8] Shisana O, Rehle T, Simbayi L, Parker W, Zuma K, Bhana A, et al. South African National HIV Prevalence, HIV Incidence, Behaviour and Communication Survey [Internet]. Cape Town; 2005. Available from: https://www.google.com/books/edition/South_African_National_HIV_Prevalence_HI/LlZRZjX-D_gC?hl=en&gbpv=1&dq=Shisana+O,+Rehle+T,+Simbayi+LC,+Parker+R,+Zuma+K,+Bhana+A,+et+al.+South+African+national+HIV+prevalence,+HIV+incidence,+behaviour+and+communication+

[9] Shisana O, Rehle T, Simbayi L, Zuma K, Jooste S, Pillay-van-Wyk V, et al. South African National HIV Prevalence, Incidence, Behaviour and Communication Survey, 2008. A Turning Tide Among Teenagers? 2009.

[10] Shisana O, Rehle T, Simbayi L., Zuma K, Jooste S, Zungu N, et al. South African National HIV Prevalence, Incidence and Behaviour Survey, 2012 [Internet]. Cape Town; 2014. Available from: https://www.hsrc.ac.za/uploads/pageContent/4565/SABSSMIVLEOfinal.pdf 10.2989/16085906.2016.1153491 27002359

[11] Simbayi L, Zuma K, Zungu N, Moyo S, Marinda E, Jooste S. South African National HIV Prevalence, Incidence and Behavior Survey, 2017. Cape Town. 2019.

[12] Human Sciences Research Council. SABSSM VI [Internet]. 2023 [cited 2023 Aug 17]. Available from: https://hsrc.ac.za/special-projects/sabssm-vi//

[13] Moyo S, Ismail F, Van der Walt M, Ismail N, Mkhondo N, Dlamini S, et al. Prevalence of bacteriologically confirmed pulmonary tuberculosis in South Africa, 2017-19: a multistage, cluster-based, cross-sectional survey. Lancet Infect Dis. 2022;22(8):1172–80. doi: 10.1016/S1473-3099(22)00149-9 35594897 PMC9300471

[14] Chanda-Kapata P, Kapata N, Klinkenberg E, Grobusch MP, Cobelens F. The prevalence of HIV among adults with pulmonary TB at a population level in Zambia. BMC Infect Dis. 2017;17(1):236. doi: 10.1186/s12879-017-2345-5 28356081 PMC5372321

[15] Matji R, Maama L, Roscigno G, Lerotholi M, Agonafir M, Sekibira R, et al. Policy and programmatic directions for the Lesotho tuberculosis programme: Findings of the national tuberculosis prevalence survey, 2019. PLoS One. 2023;18(3):e0273245. doi: 10.1371/journal.pone.0273245 36893175 PMC9997977

[16] World Health Organization. Tuberculosis prevalence surveys: a handbook. Tuberculosis. 2011.

[17] CDC Division of Global HIV & TB. PHIA surveys: a new approach to help countries control their hiv epidemics [Internet]. 2018 [cited 2023 Feb 1]. Available from: https://phia.icap.columbia.edu/wp-content/uploads/2018/10/MPHIA-SS_2018_FINAL.pdf

[18] National Department of Health. The First National TB Prevalence Study: South Africa 2018. 2020.

[19] Zuma K, Zungu N, Moyo S, Marinda E, Simbayi L, Jooste S. The Sixth South African National HIV Prevalence, Incidence and Behaviour Survey, 2022: A Summary Report. 2024.

[20] National Department of Health. 2023 ART Clinical Guidelines for the Management of HIV in Adults, Pregnancy and Breastfeeding, Adolescents, Children, Infants and Neonates. 2023.

[21] Patel HK, Duong YT, Birhanu S, Dobbs T, Lupoli K, Moore C, et al. A Comprehensive Approach to Assuring Quality of Laboratory Testing in HIV Surveys: Lessons Learned From the Population-Based HIV Impact Assessment Project. J Acquir Immune Defic Syndr. 2021;87(Suppl 1):S17–27. doi: 10.1097/QAI.0000000000002702 34166309 PMC10966620

[22] Centers for Disease Control and Prevention Division of Global HIV and TB. Implementing and Scaling up Undetectable = Untransmittable (U=U): A Resource Guide [Internet]. Atlanta, GA; 2024. Available from: https://www.cdc.gov/global-hiv-tb/media/pdfs/2024/07/U-U-Resource-Guide-July-12-2024.pdf

[23] Siddiqi SH, Rüsch-Gerdes S. MGIT Procedure Manual: For BACTEC MGIT 960 TB System. 2006;(July):1–52. Available from: http://www.finddiagnostics.org/export/sites/default/resource-centre/find_documentation/pdfs/mgit_manual_nov_2007.pdf

[24] Samodien E, Abrahams Y, Muller C, Louw J, Chellan N. Non-communicable diseases - A catastrophe for South Africa. S Afr J Sci. 2021;117(5):1–6.

[25] Law I, Floyd K, African TB Prevalence Survey Group. National tuberculosis prevalence surveys in Africa, 2008-2016: an overview of results and lessons learned. Trop Med Int Health. 2020;25(11):1308–27. doi: 10.1111/tmi.13485 32910557 PMC8043149

[26] Sachathep K, Radin E, Hladik W, Hakim A, Saito S, Burnett J, et al. Population-Based HIV Impact Assessments Survey Methods, Response, and Quality in Zimbabwe, Malawi, and Zambia. J Acquir Immune Defic Syndr. 2021;87(Suppl 1):S6–16. doi: 10.1097/QAI.0000000000002710 34166308 PMC8650710

[27] Chamie G, Clark TD, Kabami J, Kadede K, Ssemmondo E, Steinfeld R, et al. A hybrid mobile approach for population-wide HIV testing in rural east Africa: an observational study. Lancet HIV. 2016;3(3):e111-9. doi: 10.1016/S2352-3018(15)00251-9 26939734 PMC4780220

[28] Hewett PC, Erulkar AS, Mensch BS. The feasibility of computer-assisted survey interviewing in Africa: Experience from two rural districts in Kenya. 2003. 10.1177/0894439304263114

[29] World Health Organization. Consolidated guidance on tuberculosis data generation and use. Module 3. National tuberculosis prevalence surveys [Internet]. Geneva; 2025. Available from: https://www.who.int/publications/i/item/9789240108004

[30] ICAP at Columbia University. From Arm to Freezer: PHIA Project Laboratory Network Supports Ambitious HIV Testing Targets [Internet]. 2016 [cited 2023 Sep 23]. Available from: https://phia.icap.columbia.edu/from-arm-to-freezer-phia-project-laboratory-network-supports-ambitious-hiv-testing-targets//

[31] Floyd S, Sismanidis C, Yamada N, Daniel R, Lagahid J, Mecatti F, et al. Analysis of tuberculosis prevalence surveys: new guidance on best-practice methods. Emerg Themes Epidemiol. 2013;10(1):10. doi: 10.1186/1742-7622-10-10 24074436 PMC3853155

[32] ICAP at Columbia University. PHIA PROJECT | Population-based HIV Impact Assessment - Guiding the Global HIV Response [Internet]. [cited 2023 Feb 2]. Available from: https://phia.icap.columbia.edu/about//

