# Supplementary material for: Piloting the feasibility of a population-based joint TB-HIV survey in KwaZulu-Natal Province, South Africa, 2019
Source: PLOS Glob Public Health. 2026 Jan 28;6(1):e0005804. doi: 10.1371/journal.pgph.0005804 (PMC12851483; doi:10.1371/journal.pgph.0005804)
Supplement: S1 Text — (DOCX) [file pgph.0005804.s001.docx]

**FEASIBLITY STUDY ON THE JOINT PREVELANCE TUBERCULOSIS AND HIV SURVEY**

**IN**

**KWAZULU-NATAL, SOUTH AFRICA**

# APPENDICES

## Appendix 1: Volunteer Script and Prelisting Form

**Joint Tuberculosis and HIV Pilot Survey**

**A: Volunteer script**

Good day,

My name is ………I live in this community and will be working with the team from Human Sciences Research Council and its partners conducting the Joint TB and HIV survey pilot study on the following dates(…………...). *The main purpose of this study is to find out if it is possible to do a joint (combined) TB and HIV survey.*  Your household is amongst those included in the study as indicated on this map (the volunteer will show the map to household head/member). I am one of the few people visiting your household for several reasons related to possible participation in the study. With your permission, I am going to list your household/stand number, the number of people staying in the household, their ages and gender. The purpose of this information is to assist the team that will be coming to conduct the study to determine if the selected households will enable them to reach the target (required) number of people within this area appearing on the map. I will also later return with one of the team members who will be asking more details about your household and issuing/giving out invitations so that you are able to visit the hub/site (name of the site), where more study procedures will be completed.

**B: Prelisting form**

**Administrative Information**

| **Name of volunteer:** |  |
| --- | --- |
| **Telephone number of volunteer:** |  |

**Geographic information**

| **Province:** |  |
| --- | --- |
| **District:** |  |
| **Cluster/Small area layer (SAL) number:** |  |
| **Total number of households at this visiting point:** |  |

**Household listing**

| **Household number:** | | | |  | |
| --- | --- | --- | --- | --- | --- |
| **Address/Street Name/Description** | | | |  | |
| **Language (s) most often spoken in this household** | | | |  | |
| **Person number** | **Sex (M/F)** | **Relationship to household head**  **(see codes*)** | **Age (years) or indicate months for babies under 12 months** | **Slept at least 5 nights in **HH in the last Two weeks (Y/N) (Y/N)** | **Eligible**  **(Y/N)** |
| **1** |  | **Household head** |  |  |  |
| **2** |  |  |  |  |  |
| **3** |  |  |  |  |  |
| **4** |  |  |  |  |  |
| **5** |  |  |  |  |  |
| **6** |  |  |  |  |  |
| **7** |  |  |  |  |  |
| **8** |  |  |  |  |  |

***1. Spouse; 2. Child; 3. Sibling (Brother/sister); 4. Parent (mother/father); 5. Nephew/niece; 6. Other relative, **HH: Household**

**Summary**

| **Total number of people in the HH** |  |
| --- | --- |
| **Number of people aged ≥ 15 years in HH** |  |
| **Number of people aged <15 years living in HH** |  |
| **Number of eligible people:**  **(people who have slept in HH for 5 nights of previous 2 weeks)** |  |

### Appendix 2: Household Questionnaire

**Joint Tuberculosis and HIV Pilot Survey**

| **GEOGRAPHIC PARTICULARS** | | | | | | | | | |
| --- | --- | --- | --- | --- | --- | --- | --- | --- | --- |
| Province name |  | | | | | | | | |
| Province number | | | | | | | |  |  |
| Small Area layer number ( SAL number) | |  |  |  |  |  |  |  |  |
| **Household number** | | | | | | |  |  |  |
| Address | | | | | | | | | |

| Q01_INC- HHC | **Interviewer’s code** | **Enter the your fieldworker number here** |
| --- | --- | --- |
|  |  |  |

| **Q01_1IHHC-CON** | **Has consent been taken?** | **Yes** | **No** |
| --- | --- | --- | --- |
|  |  |  |  |

**The interview can proceed only when consent for the household has been taken and the consent form(s) duly signed.**

| **INTERVIEW DETAILS** | | | | | | | | | | |
| --- | --- | --- | --- | --- | --- | --- | --- | --- | --- | --- |
|  | | **Year** | | **Month** | | **Day** | | | **Time**  **code** | **Response code** |
| First visit | |  |  |  |  |  | |  |  |  |
| Second visit | |  |  |  |  |  | |  |  |  |
| Third visit | |  |  |  |  |  | |  |  |  |
| ***Final response code*** | | | | | | | | | |  |
|  | | | | | | | | | | |
| **Time code**  1 = Morning till 12:00  2 = 12:00-16:00  3 = 16:00-18:00  4 = 18:00-20:00  5 = 20:00 and later | **Response code**  1 = Interview completed  2 = Not a valid visiting point  3 = No one living here (unoccupied)  4 = Refusal by household head  5 = Refusal by other resident | | | | | | 6 = Partly completed / appointment made  7 = No one at home  8 = No one eligible to complete questionnaire  9 = Incapacitated  10 = Other | | | |

| **REFUSAL PARTICULARS** | | |
| --- | --- | --- |
| NO. | QUESTIONS AND FILTERS | CODING CATEGORIES |
| 02 | At what point did the respondents refuse to take part in the survey? | AT THE GATE OR DOOR 1  AFTER EXPLANATION OF THE SURVEY…………..2  AFTER IDENTIFYING THE RESPONDENT 3  DURING THE HOUSEHOLD INTERVIEW 4  OTHER (SPECIFY) 9 |
| 03_1 | What was/were the reason(s) for the refusal? | TOO BUSY TO GRANT INTERVIEW 1  NOT AVAILABLE NOW 2  TOO LATE IN THE EVENING 3  DON’T PARTICIPATE IN SURVEYS 4  OBJECT TO TOPIC OF THE SURVEY 5  OBJECT TO PROVIDE INFORMATION OF HOUSEHOLD MEMBERS 6  DO NOT ALLOW STRANGERS ON PROPERTY 7  ENUMERATED IN THE RECENT POPULATION CENSUS…….. 8  FEAR OF BREACH OF CONFIENTIALITY……………………..9  OTHER (SPECIFY) 10 |
| 03_2 | I agree that you can record and use information on my age, sex, and level of education. | Yes………………………………………..1  No.………………………………………..2 |

| Q04_HH head | Name of the head of household |  |
| --- | --- | --- |

| Q05_NOPHH | **How many people live in the household?** (Include only those people who have slept in the house for at least 5 nights of the prior two weeks and normally live and share meals in the household) |  |
| --- | --- | --- |

| QO6_HMHHC Details of household members | | | | | | | |
| --- | --- | --- | --- | --- | --- | --- | --- |
| **Person number** | **Name of HH member** | **Age of HH member in year**s  SELECT 00 IF UNDER  1 YEAR) | **Sex**  **Male=1**  **Female =2** | **HH visit study number** | **Participant ID**  **(from barcode)** | **Eligibility****  **1 = eligible**  **2 = ineligible** | **Invitation to Hub**  **1** **= Accepted**  **2 = Declined** |
| 1  **(Household head**) |  |  |  |  |  |  |  |
| 2 |  |  |  |  |  |  |  |
| 3 |  |  |  |  |  |  |  |
| 4 |  |  |  |  |  |  |  |
| 5 |  |  |  |  |  |  |  |
| 6 |  |  |  |  |  |  |  |
| 7 |  |  |  |  |  |  |  |
| 8 |  |  |  |  |  |  |  |
| 9 |  |  |  |  |  |  |  |
| 10 |  |  |  |  |  |  |  |
| 11 |  |  |  |  |  |  |  |
| 12 |  |  |  |  |  |  |  |

***(Use extra page if needed)***

**Those that have slept in the household for at least 5 nights of the prior two weeks are eligible to participate

| **Q07 ASHHC AVAILABILITY OF SERVICES** | | | |
| --- | --- | --- | --- |
|  | QUESTIONS AND FILTERS | CODING CATEGORIES |  |
| Q07_1 | What is the main source of drinking water for members of your household? | **(SELECT 1**)  PIPED WATER (TAP) IN DWELLING………. 1  PIPED WATER (TAP) IN SITE/YARD………. 2  BOTTLED WATER 3  WATER CARRIER/TANKER………………… 4  RAIN-WATER TANK ……………… 5  BOREHOLE / WELL / SPRING……………… 6  DAM/RIVER/STREAM………… 7  PUBLIC / COMMUNAL TAP …………………. 8  OTHER 9 |  |
| Q07_2 | What is this household’s main source of energy for cooking purposes? | **(SELECT 1**)  ELECTRICITY 1  COAL 2  WOOD 3  GAS 4  PARAFFIN 5  ANIMAL DUNG 6  OTHER 9 |  |
| Q07_3 | Does your household have any of the following (in a working condition)? (READ OPTIONS)  Electricity?  A radio?  A television?  A landline telephone  A cellphone?  A refrigerator?  A personal computer (PC)?  A washing machine? | **(SELECT THE RELEVANT OPTION**)  YES NO  ELECTRICITY 1 2  RADIO 1 2  TELEVISION 1 2  TELEPHONE (landline) 1 2  CELLPHONE 1 2  REFRIGERATOR 1 2  PERSONAL COMPUTER 1 2  WASHING MACHINE 1 2 |  |
| Q07_4 | How many rooms does your dwelling consist of?  NOTE: EXCLUDE BATHROOMS AND TOILETS. | **(SELECT VALUE**)  ┌──┬──┐  ROOMS │░ │░░│  └──┴──┘ |  |
| Q07_5 | How many rooms in your dwelling are used for sleeping?  NOTE: A ROOM MAY ALSO HAVE ANOTHER PURPOSE BESIDES AS A BEDROOM. | **(SELECT VALUE**)    ┌──┬──┐  ROOMS FOR SLEEPING │░ │░░│  └──┴──┘ |  |

| **Q08 IV HHCINDICATORS OF VULNERABILITY** | | |
| --- | --- | --- |
| Q08_1 | **SELECT ONLY ONE OPTION**  The cost of living is a concern for many families. Can you tell me what this household can afford? Which option best describes your household situation? | NOT ENOUGH MONEY FOR BASIC THINGS LIKE FOOD AND CLOTHES…………………………… 1  MONEY FOR FOOD AND CLOTHES, BUT SHORT ON MANY OTHER THINGS…………………………….2  WE HAVE MOST OF THE IMPORTANT THINGS, BUT FEW LUXURY GOODS……………………………..3  MONEY FOR EXTRA THINGS SUCH AS HOLIDAYS AND LUXURY GOODS………………………………4 |
| Q08_2 | Can you please tell me what the approximate monthly income is for this household? | (**ENTER THE AMOUNT REPORTED)**  1. R . .  2. not known/ did not answer |
| Q08_3 | **SELECT ONLY ONE OPTION**  In the past 4 weeks, was there ever no food to eat (of any kind) in your household, because of a lack of resources to get food? | YES 1  NO 2  DON’T KNOW 3  **(If “No” or ‘Don’t know” conclude the interview )** |
| Q08_4 | **SELECT ONLY ONE OPTION**  How often did this happen in the past 4 weeks? | RARELY (1-2 TIMES) 1  SOMETIMES (3-10 TIMES)………….. 2  OFTEN (MORE THAN 10 TIMES)………... 3 |
| Q08_5 | **SELECT ONLY ONE OPTION**  In the past 4 weeks, did you or any household member go to sleep at night hungry because there was not enough food? | YES 1  NO 2  DON’T KNOW 3  **(If “No” or ‘Don’t know” –conclude the interview)** |
| Q08_6 | **SELECT ONLY ONE OPTION**  How often did this happen in the past 4 weeks? | RARELY (1-2 TIMES) 1  SOMETIMES (3-10 TIMES)………….. 2  OFTEN (MORE THAN 10 TIMES)………... 3 |

**INSTRUCTION:** BEFORE LEAVING THE HOUSEHOLD ENSURE THAT

- **ALL ELIGIBLE INDIVIDUALS WHO HAVE AGREED TO PARTICIPATE HAVE A HUB APPOINTMENT CARD**
- **ALL ELIGIBLE INDIVIDUALS WHO HAVE AGREED TO PARTICIPATE ARE AWARE OF THE TIME AT WHICH THEY SHOULD PRESENT AT THE SITE**

**BOX 2: INTERVIEWERS’ SCRIPT TO EXPLAIN EXCLUSION OF INELIGIBLEELIGIBLE INDIVIDUALS**

We have a checklist which enables people to be allowed to be part of the survey. Part of the checklist includes reviewing the number of days you have spent/slept at home. We also consider age for your children to be screened for TB. Unfortunately, based on the checklist and the information you provided, we are not able to allow to be part of the study. Should you have any concerns about your health and desire to be tested for HIV, we encourage you to go to your nearest clinic.

## Appendix 3: Barcoded Invitation Form Card

Principal Investigator: Dr Sizulu Moyo (Human Sciences Research Council)

Funding source: The US President’s Emergency Plan for AIDS Relief (PEPFAR)

through the US Centers for Disease Control and Prevention (CDC)

(Cooperative Agreement #GH001629),

***Joint Tuberculosis and HIV Pilot Survey***

**Flesh-Kincaid Level = 7.6**

Good day,

The Human Sciences Research Council (HSRC) in collaboration with other research organisations is conducting a Joint Tuberculosis (TB) and HIV Pilot Survey, also called the combined TB/HIV pilot survey. The main purpose of this study is to find out if it is possible to do a joint (combined) TB and HIV survey in South Africa. You are being invited to take part in the survey activities that will be conducted at the survey hub that is located at ……………………………………………(place) from ………(day)/………(month)./2019.You are invited because you agreed that you slept in this home for 5 nights of the last two weeks.. At the hub you will be asked questions about your health including questions about HIV and TB. We will measure your weight how heavy you are), your height (how tall you are), your blood pressure (BP) blood glucose (amount of sugar in the blood), and cholesterol (amount of fat in the blood) if you are 18 years and older. This will provide opportunity for you to know about some aspects of your health. You will also be asked to give a blood sample for an HIV test, and other HIV relates tests that are viral load (the amount of the HIV in the blood), recency of HIV infection (to see if infection with HIV happened recently),,and resistance to antiretroviral drugs (ARVs) (whether the HIV drugs work well against the HIV). You will also be asked to take a chest X-ray, and if necessary, to give a sputum sample (phlegm) to be tested for TB. A chest X-ray and sputum sample will be taken from those who are 15 years and older. If your children are taking part, we will ask you questions about their health, if they are younger than 12 years. Those who are 12 years and older will be asked to answer questions on their own. We will also collect blood from the children for HIV testing if they agree. This will provide the opportunity to know one’s HIV status, including that of children. Children will also be given opportunity to indicate whether they agree to give a blood sample. All the work will be done by specially trained people. Taking part in the study is strictly voluntary. We invite you to visit the hub, where you will be provided with more details about the study. You will receive a small packet of household items or airtime as reimbursement for your time. Children will also a small packet of household items or child friendly items, or airtime as reimbursement for their time spent on the study.

Your appointment has been scheduled as follows:

**Date:…………/…………………/………………….:**

**Time:……………………………………… (**You are also free to come at another time that is convenient to you between …………/………/2019 and …………/…………/2019)

*Please keep this slip safely, and bring it when you come to our site. We thank you for your time.*

| **Participant** | **Name** | **Surname** | **Age** | **Sex** | **Barcode** |
| --- | --- | --- | --- | --- | --- |
| Adult Participant |  |  |  |  |  |
| Accompanying child 1 |  |  |  |  |  |
| Accompanying child 2 |  |  |  |  |  |
| Accompanying child 3 |  |  |  |  |  |
| Accompanying child 4 |  |  |  |  |  |
| Accompanying child 5 |  |  |  |  |  |

**Add more rows if there are more than 5 children**

| D | D | M | M | Y | Y | Y | Y |
| --- | --- | --- | --- | --- | --- | --- | --- |

**Date the invitation is given**

**Field Staff member’s name:**

## Appendix 4: Contact Details Form

***Joint Tuberculosis and HIV Pilot Survey***

**Geographic information**

| **Province:** |  |
| --- | --- |
| **District:** |  |
| **Cluster/SAL number:** |  |

|  | **Barcode** | **Sex** | **Age** | **address** | **Phone number** | **Reason for details (self or parent/ guardian** |
| --- | --- | --- | --- | --- | --- | --- |
|  |  |  |  |  |  |  |
|  |  |  |  |  |  |  |
|  |  |  |  |  |  |  |
|  |  |  |  |  |  |  |
|  |  |  |  |  |  |  |
|  |  |  |  |  |  |  |
|  |  |  |  |  |  |  |
|  |  |  |  |  |  |  |
|  |  |  |  |  |  |  |
|  |  |  |  |  |  |  |
|  |  |  |  |  |  |  |
|  |  |  |  |  |  |  |

## Appendix 5: Individual Questionnaires

### **A: Individual Questionnaire for persons aged 15 years and older (Brief version)**

***Joint Tuberculosis and HIV Pilot Survey***

Barcode

| **A** | **GEOGRAPHIC AND INTERVIEW PARTICULARS** | | | | | | | | |
| --- | --- | --- | --- | --- | --- | --- | --- | --- | --- |
| Province | | | | | | | | |  |
| Cluster number | | | | |  |  |  |  |  |
| Small area layer | |  |  |  |  |  |  |  |  |
| Visiting point number (from the map) | | | | |  |  |  |  |  |
| Person number of respondent | | | | | | | |  |  |

| **B** | **INTERVIEW DETAILS** | | | | | | | | |
| --- | --- | --- | --- | --- | --- | --- | --- | --- | --- |
|  | | Year | | Month | | Day | | Time code | Response code |
| First visit | | 1 |  |  |  |  |  |  |  |
| Second visit | | 1 |  |  |  |  |  |  |  |
| Third visit | | 1 |  |  |  |  |  |  |  |
| Final response code | | | | | | | | |  |
| **Time code**  1 = Morning till 12:00  2 = 12:01-16:00  3 = 16:01-18:00  4 = 18:01-20:00  5 = 20:01 and later | | **Response code**  1 = Interview completed and sample taken  2 = Interview completed but sample not taken  3 = Appointment made for interview and/or sample  4 = Selected respondent not at home  5 = Refusal by head of household  6 = Refusal by respondent  7 = Other | | | | | | | |

| **INTERVIEW STARTING TIME:** |  |  |  |  |  |
| --- | --- | --- | --- | --- | --- |

| **INTERVIEWER: NAME AND EMPLOYEE NUMBER**  **…………………………………………………………..…………………..** |  |  |  |  |  |  |  |
| --- | --- | --- | --- | --- | --- | --- | --- |

| **C** | **REFUSAL PARTICULARS (IF APPLICABLE)** | |
| --- | --- | --- |
| At what point did the respondent refuse?  SPECIFY | |  |

1 = At the reception desk

2 = After explanation of the survey and the process (group information session)

4 = During the individual interview

5 = After the individual interview when requested to do the test

6 = Other

Refusals during individual interview

20 = Objected to providing any/some information on the topic

21 = Objected to providing personal/confidential information

22 = Unable to provide requested information

23 = Refused to continue because he/she got irritated/bored

24 = Refused to continue because he/she got angry

25 = Refused to continue because he/she lost interest or got tired

26 = Refused to continue because he/she was in a hurry

27 = Other

Refusal to provide a blood sample

40 = Apprehensive of blood sample being taken

41 = Against religious beliefs to provide a blood sample

42 = Did not want to know HIV status

43 = Fear a breach of confidentiality

44 = Did not trust the interviewers

45 = Recently had an HIV test

46 = Did not to disclose status to parents/guardian

47 = Other

Refusal to take a chest –X-ray

48 = Apprehensive of taking the CXR image

49 = Against religious beliefs to take CXR

50 =-pregnant

51 = afraid

| Collection and use of demographic information after refusal to participate or withdrawal from the study | | |
| --- | --- | --- |
|  | I agree that you can record and use information on my age, sex, and level of education. | Yes………………………………………..1  No.………………………………………..2 |

| **GENERAL INSTRUCTION** | **CIRCLE THE CODE NEXT TO THE APPROPRIATE ANSWER. IF INDICATED READ THE ANSWER OPTIONS. *Please remember that your name is not written anywhere and everything you tell me is confidential.*** |
| --- | --- |

| **SECTION 1** | **RESPONDENT’S BIOGRAPHICAL DATA** |
| --- | --- |

| **1.1** | **How old were you on your last birthday? (*Age of the respondent*)** | | | | |  | |  | |
| --- | --- | --- | --- | --- | --- | --- | --- | --- | --- |
| **1.1.1** | **What is your date of birth?** | | | | | | | | |
| Year | | | Month | |  | | | |  |
|  | |  |  |  |  | |  | |  |

| **INSTRUCTION** | **DO NOT ASK; RECORD SEX** | Male | Female |
| --- | --- | --- | --- |
| **1.2** | **Sex of the respondent** | 1 | 2 |

| **INSTRUCTION** | **READ EACH OPTION** |
| --- | --- |

| **INSTRUCTION** | **How would you describe yourself in terms of gender?** | Male | Female | Transgender |
| --- | --- | --- | --- | --- |
| **1.3** |  | 1 | 2 | 3 |

| **INSTRUCTION** | | **Which of the following describes your race?** | | | |
| --- | --- | --- | --- | --- | --- |
| **1.3a** | |  |  |  |  |
| African | White | | Coloured | Indian/Asian | Other |
| 1 | 2 | | 3 | 4 | 5 |

| **INSTRUCTION** | ***I am now going to ask you about your marital status*** |
| --- | --- |

| **1.4a** | **What is your current marital status? (Marital status referring to legal, traditional or common-law)** | |
| --- | --- | --- |
| Married | | 1 |
| Never married *GO TO 1.4c* | | 2 |
| Divorced/separated | | 3 |
| Widower/widow | | 4 |

| **1.4b** | **How old were you when you were married for the first time?** |  |  |
| --- | --- | --- | --- |

| **INSTRUCTION** | **READ EACH OPTION** |
| --- | --- |

| **1.4c** | **What is your current living arrangement?** | |
| --- | --- | --- |
| Living with husband/wife | | 1 |
| Living on own or other arrangement but not living with husband/wife | | 2 |
| Living together with boyfriend/girlfriend/civil union (same sex) partner/other partner | | 3 |
| Single/divorced/widowed – in a steady relationship but not living together | | 4 |
| Single; not in a steady relationship | | 5 |

| **MARRIED RESPONDENTS** | **RESPONDENTS NOT MARRIED**  **Q1.7** |
| --- | --- |

| **1.5** | | **Are you in a polygamous union?**  **(**the practice or custom of having more than one wife or husband at the same time) | Yes | No |
| --- | --- | --- | --- | --- |
|  |  |  | 1 | 2 |
|  |  | | | ***GO TO 1.7*** |

| **1.6** | **Altogether, how many wives do you have/or how many wives does your husband have?** |  |  |
| --- | --- | --- | --- |

| **INSTRUCTION** | ***I am now going to ask about your employment situation*** |
| --- | --- |

| **1.7** | **How would you describe your present employment situation?** | |
| --- | --- | --- |
| Unemployed | | 1 |
| Sick/disabled and unable to work | | 2 |
| Student/pupil/learner | | 3 |
| Employed/Self employed | | 4 |
| Other | | 5 |

| **1.8** | **Did you receive an income from any source in the last month?** | Yes | No |
| --- | --- | --- | --- |
|  |  | 1 | 2 |
|  |  | | ***GO TO 1.11*** |

| **1.9** | **What was your main source of income in the last month?** | |
| --- | --- | --- |
| Salary/earnings | | 1 |
| Contributions by family members or relatives | | 2 |
| Government pensions/grants (e.g., old age pension, child support grant, disability grant) | | 3 |
| Grants/donations by private welfare organizations | | 4 |
| Other sources (Specify?.......................................................) | | 5 |

| **1.9a** | **If employed (part time, occasional, seasonal or full-time), in what sector was this in?** | |
| --- | --- | --- |
| Mining | | 1 |
| Health Care | | 2 |
| Correctional services | | 3 |
| Agriculture | | 4 |
| Other sources (Specify?........................................................) | | 5 |

| **1.10** | **What is your gross monthly income? R** |  |  |  |  |  |  |
| --- | --- | --- | --- | --- | --- | --- | --- |

| **1.11** | **Do you have a disability?** | Yes | No | Don’t know |
| --- | --- | --- | --- | --- |
|  |  | 1 | 2 | 3 |
|  |  | | ***GO TO 1.14*** | ***GO TO 1.14*** |

| **1.12** | **How long have you had the disability?**  **FILL IN ‘00’IF LESS THAN ONE YEAR** | Years | |
| --- | --- | --- | --- |
|  |  |  |  |

| **INSTRUCTION** | **DO NOT READ OUT OPTIONS, MULTIPLE RESPONSES POSSIBLE** |
| --- | --- |

| **1.13** | **What is the disability?** | |
| --- | --- | --- |
| **a** | Physical (spinal injury, loss of a limb, etc.) | 1 |
| **b** | Sight | 2 |
| **c** | Partial hearing | 3 |
| **d** | Communication/speech | 4 |
| **e** | Mental or psychiatric illness | 5 |

| **INSTRUCTION** | **SCHOOL ATTENDANCE** |
| --- | --- |

| **1.14** | **Have you ever attended school?** | Yes | No |
| --- | --- | --- | --- |
|  |  | 1 | 2 |
|  |  | | ***GO TO 2.1*** |

| **1.15a** | **Are you currently attending school/post-school?** | Yes | No |
| --- | --- | --- | --- |
|  |  | 1 | 2 |
|  |  | | ***GO TO 1.15c*** |

| **1.15b** | **What grade are you attending this year?** | |
| --- | --- | --- |
| Grade 3/Standard 1/Abet 1 | | 3 |
| Grade 4/Standard 2/Abet 2 | | 4 |
| Grade 5/Standard 3/Abet 2 | | 5 |
| Grade 6/Standard 4/Abet 3 | | 6 |
| Grade 7/Standard 5/Abet 3 | | 7 |
| Grade 8/Standard 6/Abet 3 | | 8 |
| Grade 9/Standard 7Abet 3 | | 9 |
| Grade 10/Standard 8/Ntc 1 | | 10 |
| Grade 11/Standard 9/Ntc 2 | | 11 |
| Grade 12/Standard 10/Ntc 3 | | 12 |
| Diploma/undergraduate degree/other post school | | 13 |
| Further degree | | 14 |
|  | | ***GO 2.1*** |

| **1.15c** | **What is the highest educational level that you obtained?** | |
| --- | --- | --- |
| Pre-school/ Gr R | | 0 |
| Grade 1/Sub a/Class 1 | | 1 |
| Grade 2/Sub b/Class 2 | | 2 |
| Grade 3/Standard 1/Abet 1 | | 3 |
| Grade 4 /Standard 2/Abet 2 | | 4 |
| Grade 5 /Standard 3/Abet 2 | | 5 |
| Grade 6 /Standard 4/Abet 3 | | 6 |
| Grade 7/Standard 5/Abet 3 | | 7 |
| Grade 8 /Standard 6/Abet 3 | | 8 |
| Grade 9 /Standard 7/Abet 3 | | 9 |
| Grade 10/Standard 8/Ntc 1 | | 10 |
| Grade 11/Standard 9/Ntc 2 | | 11 |
| Grade 12/Standard 10/Ntc 3 | | 12 |
| Further studies incomplete | | 13 |
| Diploma/undergraduate degree/other post school completed | | 14 |
| Further degree completed | | 15 |
| Don’t know | | 98 |

| **SECTION 2** | **KNOWLEDGE AND PERCEPTIONS OF HIV/AIDS.** |
| --- | --- |

| **INSTRUCTION** | ***I am now going to ask you question about your knowledge and perceptions of HIV/AIDS Please remember that your name is not written anywhere and everything you tell me is confidential*** |
| --- | --- |

| **INSTRUCTION**  **2.2** | | ***I am now going to ask you a number of additional questions about knowledge and perceptions of HIV and AIDS*** | **Yes** | **No** | **Don’t know** |
| --- | --- | --- | --- | --- | --- |
| **a** | Can AIDS be cured? | | 1 | 2 | 3 |
| **b** | Can a person reduce the risk of HIV by having fewer sexual partners? | | 1 | 2 | 3 |
| **c** | Can a healthy-looking person have HIV? | | 1 | 2 | 3 |
| **d** | Can HIV be transmitted from a mother to her unborn baby? | | 1 | 2 | 3 |
| **e** | Can the risk of HIV transmission be reduced by having sex with only one uninfected partner who has no other partners? | | 1 | 2 | 3 |
| **f** | Can a person get HIV by sharing food with someone who is infected? | | 1 | 2 | 3 |
| **g** | Can a person reduce the risk of getting HIV by using a condom every time he/she has sex? | | 1 | 2 | 3 |
| **h** | Can medical male circumcision reduce the risk of HIV infection in males? | | 1 | 2 | 3 |
| **i** | Can the risk of HIV transmission through sex be reduced by a HIV-positive partner consistently taking drugs that treat HIV? | | 1 | 2 | 3 |

| **INSTRUCTION** | | | ***Now I want to ask you some questions relating to people living with HIV/AIDS*** | **Yes** | **No** | **Don’t know** |
| --- | --- | --- | --- | --- | --- | --- |
| **2.5** | |  |  |  |  |  |
| **a** | If you knew that a shopkeeper or food seller had HIV, would you buy food from them? | | | 1 | 2 | 3 |
| **b** | Would you buy fresh vegetables from a shopkeeper or vendor if you knew that this person had HIV? | | | 1 | 2 | 3 |
| **c** | Would you be willing to care for a family member with AIDS? | | | 1 | 2 | 3 |
| **d** | If a teacher has HIV but is not sick, should he or she be allowed to continue teaching? | | | 1 | 2 | 3 |
| **e** | Is it a waste of money to train or give a promotion to someone with HIV/AIDS? | | | 1 | 2 | 3 |
| **f** | Would you want to keep the HIV-positive status of a family member a secret? | | | 1 | 2 | 3 |
| **h** | Are you comfortable talking to at least one member of your family about HIV/AIDS? | | | 1 | 2 | 3 |
| **i** | A person would be foolish to marry a person who is living with HIV/AIDS | | | 1 | 2 | 3 |
| **j** | If a pupil has HIV but not sick, should he or she be allowed to continue to go to school? | | | 1 | 2 | 3 |
| **k** | Do you think children living with HIV should be able to attend school with children who are HIV negative? | | | 1 | 2 | 3 |

| **SECTION 3** | **I WILL NOW ASK YOU ABOUT TB *Please remember that your name is not written anywhere and everything you tell me is confidential.*** |
| --- | --- |

| **3.1** | **Have you ever heard about TB?** | **Yes** | **No** |
| --- | --- | --- | --- |
|  |  | 1 | 2 |

|  | **3.2** | **Have you ever been diagnosed with TB** | **Yes** | **No** |
| --- | --- | --- | --- | --- |
|  |  |  | 1 | 2 |
|  | | | | ***GO 3.8*** |

| **3.3** | **Where did you first present your symptoms** | |
| --- | --- | --- |
| **a** | Government/community clinic | 1 |
| **b** | Private Clinic /hospital | 2 |
| **c** | Government Provincial/District hospital | 3 |
| **d** | Private Pharmacy | 4 |
| **e** | Private Doctor | 5 |
| **f** | Traditional Healer | 6 |
| **g** | Other | 7 |

|  | **Current TB treatment** |
| --- | --- |

| **3.4** | **Are you currently on TB treatment?**  Probe and be sure the participant only refers to conventional treatment for TB | **Yes** | **No** |
| --- | --- | --- | --- |
|  |  | 1 | 2 |

| **3.5** | **For how long have you been on TB treatment** |  | months |
| --- | --- | --- | --- |
|  |  | 99 |  |

| **3.6** | **Did you complete your TB treatment, i.e., were you informed by a nurse or doctor that you no longer needed to take treatment for TB?** | **Yes** | **No** | **Still on treatment** |
| --- | --- | --- | --- | --- |
|  |  | 1 | 2 | 3 |

| **3.7** | **When you fell sick with TB, were you:** | **Yes** | **No** |
| --- | --- | --- | --- |
| **a** | Teased, insulted or sworn at? | 1 | 2 |
| **b** | Gossiped about? | 1 | 2 |
| **c** | Did you feel unclean or dirty because of your TB? | 1 | 2 |
| **d** | Did you tell anyone outside your household about your TB diagnosis? | 1 | 2 |

| **3.8** | ***Now I want to ask you some questions relating to how TB can be cured*** | **Yes** | **No** | **Don’t Know** |
| --- | --- | --- | --- | --- |
| **a** | Can TB be cured by herbal remedies? | 1 | 2 | 3 |
| **b** | Can someone with TB be cured by home rest without any medicine? | 1 | 2 | 3 |
| **c** | Can TB be cured by traditional or religious practices? | 1 | 2 | 3 |
| **d** | Can TB be cured by specific drugs given by a health professional that are taken for the entire recommended duration | 1 | 2 | 3 |
| **e** | Can TB be cured in people with HIV? | 1 | 2 | 3 |

|  | **Previous treatment** |
| --- | --- |

| **3.10** | How many times have you been on TB treatment before? (excludes current episode for those who are currently on treatment for TB) | |
| --- | --- | --- |
| Once | | 1 |
| Twice | | 2 |
| Three times | | 3 |
| More than three times | | 4 |
| Don’t know | | 5 |

| **3.11** | In your last TB episode for how many months did you take treatment? (excludes current episode for those who are currently on treatment for TB) | months | Don’t know/can’t remember |
| --- | --- | --- | --- |
|  |  |  | 999 |

| **3.12** | **Did you complete your TB treatment, i.e., were you informed by a nurse or doctor that you no longer needed to take treatment for TB?** | **Yes** | **No** | **Still on treatment** |
| --- | --- | --- | --- | --- |
|  |  | 1 | 2 | 3 |

| **3.13** | What type of TB were you treated for? | |
| --- | --- | --- |
| **a** | Normal TB (drug sensitive TB) | 1 |
| **b** | MDR or XDR TB | 2 |
| **c** | Don’t Know /Can’t remember | 999 |

| **3.14** | Have you ever been on preventive therapy for TB (IPT)? | No | Yes | Don’t Know/  can’t remember |
| --- | --- | --- | --- | --- |
|  |  | 1 | 2 | 999 |

| **INSTRUCTION** | ***I will now ask you about your state of health*** |
| --- | --- |

|  | **3.15** | **Do you currently have a cough?** | Yes | | No | |
| --- | --- | --- | --- | --- | --- | --- |
|  |  |  | 1 |  | 2 |  |
|  | | | |  | ***GO TO 3.17*** |  |

| **3.16** | For how long have you been coughing? (probe ) | |
| --- | --- | --- |
| <1 week | | 1 |
| 1-2 weeks | | 2 |
| ≥2 weeks | | 3 |
| Don’t know/ can’t remember | | 999 |

|  | **3.17** | Do you currently have a fever? (probe) | | No | Yes | Don’t Know |
| --- | --- | --- | --- | --- | --- | --- |
|  | a |  | | 1 | 2 | 999 |
|  | | |  | | ***GO TO***  ***3.19*** |  |

| **3.18** | How long have you had this fever? (probe) | <2weeks | ≥2weeks | Don’t Know |
| --- | --- | --- | --- | --- |
| A |  | 0 | 1 | 999 |

| **3.19** | Do you currently have drenching night sweats? (probe)  (So that you have to change bedding or nightclothes? | **Yes** | **No** |
| --- | --- | --- | --- |
|  |  | 1 | 2 |

| **3.20** | In the last month have you lost weight unintentionally? (probe) | No | Yes | Don’t Know |
| --- | --- | --- | --- | --- |
|  |  | 1 | 2 | 999 |

| **INSTRUCTION** | **If no to 3.16, 3.17, 3.18, 3.19, 3.20 go to 3.29** |
| --- | --- |

| **3.21** | Did you consult anybody for any of these symptoms | No | Yes | N/A |
| --- | --- | --- | --- | --- |
|  |  | 1 | 2 | 999 |
| **a** | Cough | 1 | 2 | 999 |
| **b** | Fever | 1 | 2 | 999 |
| **c** | Night sweats | 1 | 2 | 999 |
| **d** | Unintentional weight loss | 1 | 2 | 999 |

| **INSTRUCTION** | **If no for all go to 3.29** |
| --- | --- |

| **3.22** | Where did you go for help first? | |
| --- | --- | --- |
| Government/Community clinic | | 1 |
| Private Clinic/hospital | | 2 |
| Government Provincial/District hospital | | 3 |
| Private Pharmacy | | 4 |
| Private Doctor | | 5 |
| Traditional Healer | | 6 |
| Other | | 7 |

| **3.23** | If private clinic or hospital or doctor/ pharmacy /tradition healer, did you ever go to a government/community/sputum collection point and give a sputum sample for lab testing? | **Yes** | **No** |
| --- | --- | --- | --- |
|  |  | 1 | 2 |

| **3.24** | What has been done for you when you sought help?  **(Read out the response options. Multiple responses are possible.)** | No | Yes | N/A | Don’t know |
| --- | --- | --- | --- | --- | --- |
| **a** | 1 sputum sample was collected | 1 | 2 | 3 | 4 |
| **b** | 2 sputum samples were collected | 1 | 2 | 3 | 4 |
| **c** | A CXR done | 1 | 2 | 3 | 4 |

| **INSTRUCTION** | **If no for all go to 3.29** |
| --- | --- |

| **3.25** | What were the results of the following tests | Negative for TB | Positive  For TB | Don’t know/ Did not get results | N/A |
| --- | --- | --- | --- | --- | --- |
| **a** | sputum sample collected | 1 | 2 | 3 | 4 |
| **b** | CXR | 1 | 2 | 3 | 4 |

| **3.26** | **Were you also tested for HIV at the TB clinic?** | Yes | No | Don’t know |
| --- | --- | --- | --- | --- |
|  |  | 1 | 2 | 3 |

| **3.27** | If results were positive for TB above, where you started on treatment for: ? | Yes | No | Don’t know |
| --- | --- | --- | --- | --- |
| **a** | TB | 1 | 2 | 3 |
| **b** | HIV | 1 | 2 | 3 |

| **3.28** | What are the reasons for not seeking care for any of the symptoms? **(multiple responses possible)** | |
| --- | --- | --- |
| **a** | Distance- the health centre is far from where I live | 1 |
| **b** | Money - I had no money for transport to the health centre | 2 |
| **c** | Relevance - I did not consider it to be important | 3 |
| **d** | Still planning to seek care | 4 |
| **e** | Other specify | 5 |

| **INSTRUCTION** | ***I am now going to ask you about tuberculosis (TB). Please remember that there are no correct or wrong answers*** |
| --- | --- |

| **3.29** | **Do you agree or disagree with the following statements** | **Agree** | **Disagree** | **Don’t Know** |
| --- | --- | --- | --- | --- |
| **a** | Anybody can get TB | 1 | 2 | 3 |
| **b** | People living with HIV are more likely to get  TB | 1 | 2 | 3 |
| **c** | People that are HIV negative can get TB | 1 | 2 | 3 |

| **3.30** | **Do you agree or disagree with the following statements** | **Agree** | **Disagree** | **Don’t Know** |
| --- | --- | --- | --- | --- |
| **a** | A person can prevent getting TB by avoiding shaking hands | 1 | 2 | 3 |
| **b** | A person can prevent spreading TB by covering the mouth when coughing or sneezing | 1 | 2 | 3 |
| **c** | A person can prevent getting TB by opening windows at home and in public areas | 1 | 2 | 3 |
| **d** | A person can prevent getting TB  by limiting close contact with people who have TB that is not treated | 1 | 2 | 3 |
| **e** | A person can prevent getting TB  by completing religious or traditional practices | 1 | 2 | 3 |
| **f** | A person living with HIV can prevent TB by using specific medication to prevent TB,  that is given by  health professionals | 1 | 2 | 3 |

| **3.31** | **Are people with TB always also HIV positive?** | **Yes** | **No** | **Don’t know** |
| --- | --- | --- | --- | --- |
|  |  | 1 | 2 | 3 |

| **INSTRUCTION** | **READ EACH STATEMENT AND CHECK ONE ANSWER** |
| --- | --- |

| **3.32** | **In your community, how is a person who has TB usually regarded/treated? I am going to read all options and you can tell me which one best describes your community** | |
| --- | --- | --- |
| Most people reject him or her | | 1 |
| Most people are friendly but they generally try to avoid him or her | | 2 |
| The community mostly supports him or her | | 3 |
| Do not know any person who has TB | | 4 |
| Do not know how my community treats a person who has TB | | 5 |
| Other | | 6 |

| **INSTRUCTION** | | **DO NOT READ OUT OPTIONS, MULTIPLE RESPONSES POSSIBLE** | |
| --- | --- | --- | --- |
| **3.33** | **What would be your reaction if you found out that you have TB?** | | |
| **a** | Fear | | 1 |
| **b** | Surprise | | 2 |
| **c** | Shame/ embarrassment | | 3 |
| **d** | Sadness/hopelessness | | 4 |
| **e** | Other | | 5 |

| **SECTION 4** | **SEXUAL HISTORY** |
| --- | --- |

| **INSTRUCTION** | ***I now have to ask you sensitive questions on sex and other sex-related matters. Please remember that your name will not be recorded anywhere in this questionnaire and the information you give will be kept confidential.*** |
| --- | --- |

| **4.1** | **Have you ever had sexual intercourse? [***That is when the penis is in the vagina or anus]* | Yes | No | No response |
| --- | --- | --- | --- | --- |
|  |  | 1 | 2 | 3 |
|  | | ***Go to 4.3*** |  | ***Go to 7.1*** |

| **INSTRUCTION** | **AGE AND SEX FILTER** | |
| --- | --- | --- |
| **YOUTH 15 TO 24 YEARS**  **WHO NEVER HAD SEX** | | **MEN AND WOMEN**  **7.1**  **25 YEARS AND OLDER**  **WHO NEVER HAD SEX** |

| **INSTRUCTION** | **DO NOT READ OUT OPTIONS, MULTIPLE RESPONSES POSSIBLE** |
| --- | --- |

| **4.2** | **Could you please tell me why you have not had sex yet?** | |
| --- | --- | --- |
| **a** | Not ready | 1 |
| **b** | I am too young | 2 |
| **c** | Not interested | 3 |
| **d** | Avoiding pregnancy | 4 |
| **e** | Avoiding STDs, including HIV | 5 |
| **f** | Religious grounds | 6 |
| **g** | Cultural grounds | 7 |
| **h** | Don't have a partner | 8 |
| **i** | No response | 9 |
| **j** | Other (Specify) | 10 |
|  | | ***Go to 7.1*** |

| **4.3** | **How old were you when you had sex for the first time? ______________yrs old** |  |  | Cannot remember the age |
| --- | --- | --- | --- | --- |
|  | | | | 1 |

| **4.4** | **How many people have you had sexual intercourse with in your lifetime?** | | | | |  |  |
| --- | --- | --- | --- | --- | --- | --- | --- |
| **4.5** | | **Did you use a condom the first time you had sex?** | Yes | No | Cannot remember | | |
|  |  |  | 1 | 2 | 3 | | |

| **SECTION 5** | **PARTNER(S) AND PARTNER CHARACTERISTICS** |
| --- | --- |

| **INSTRUCTION** | ***I am now going to ask you questions on partner(s) and partner characteristics. Please remember that your name will not be recorded anywhere in this questionnaire and the information you give will be kept confidential.*** |
| --- | --- |

| **5.1** | **Have you had sex during the past 12 months?** | Yes | No | No response |
| --- | --- | --- | --- | --- |
|  |  | 1 | 2 | 3 |
|  | | | ***Go to 7.1*** | ***Go to 7.1*** |

| **5.2** | **Overall, how many sexual partners did you have during the past 12 months?** | | **0** | 1 |
| --- | --- | --- | --- | --- |
| **INSTRUCTION** | | **IF ‘00’, CLARIFY THE ANSWER IN Q5.1** | | |

| **5.3** | **How many male sexual partners did you have during the past 12 months?** |  |  |
| --- | --- | --- | --- |

| **5.4** | **How many female sexual partners did you have during the past 12 months?** |  |  |
| --- | --- | --- | --- |

| **5.4a** | | **Sum answers to 5.3 and 5.4 and enter TOTAL** | |  | |  |
| --- | --- | --- | --- | --- | --- | --- |
| **INSTRUCTION** | |  |  |  |  |  |
| **5.5** | **Just to make sure that I have this right: you have had in TOTAL _______ sexual partners during the past 12 months. Is that correct?** | | **Yes** | | **No** | |
|  |  |  | 1 | | 2 | |
|  | | | | | **Probe and correct** | |

| **INSTRUCTION** | ***Now I would like to talk with you about your sexual activity in general*** |
| --- | --- |

| **5.5a** | **Have you ever received money, gifts, or favours in exchange for sex?** | **Yes** | **No** | **Don’t know** |
| --- | --- | --- | --- | --- |
|  |  | 1 | 2 | 3 |
|  |  | | ***GO TO 5.5d*** | |

| **5.5b** | **In the last 12 months, have you received money, gifts, or favours in exchange for sex?** | Yes | | No |
| --- | --- | --- | --- | --- |
|  |  | 1 | | 2 |
|  | | |  | ***GO TO 5.5d*** |

| **5.5c** | **The last time you received money, gifts, or favours in exchange for sex, was a condom used?** | **Yes** | **No** | **Don’t know** |
| --- | --- | --- | --- | --- |
|  |  | 1 | 2 | 3 |

| **5.5d** | **Have you ever given money, gifts, or favours in exchange for sex?** | **Yes** | **No** | **Refused** |
| --- | --- | --- | --- | --- |
|  |  | 1 | 2 | 3 |
|  |  | | ***GO TO NEXT FILTER*** | |

| **5.5e** | **In the last 12-months, have you given money, gifts, or favours in exchange for sex?** | **Yes** | **No** | **Refused** |
| --- | --- | --- | --- | --- |
|  |  | 1 | 2 | 3 |
|  |  | | ***GO TO NEXT FILTER*** | |

| **5.5f** | **The last time you gave money, gifts, or favours in exchange for sex, was a condom used?** | **Yes** | **No** | **Don’t know** |
| --- | --- | --- | --- | --- |
|  |  | 1 | 2 | 3 |

| **5.5g** | **Have any of these sexual partner(s) had other sexual partners in the past 12 months?** | Yes | No | Don’t Know |
| --- | --- | --- | --- | --- |
|  |  | 1 | 2 | 3 |

| **INSTRUCTION** | **SEXUAL PARTNERS FILTER** | |
| --- | --- | --- |
| **More than one sexual partner** | | **One sexual partner**  **GO TO NEXT FILTER** |

| **5.6** | **Did any of these relationships mentioned above overlap with each other?** | Yes | No | No response |
| --- | --- | --- | --- | --- |
|  |  | 1 | 2 | 3 |

| **5.7** | **Do you have two or more sexual partners at the moment?** | **Yes** | **No** | **No response** |
| --- | --- | --- | --- | --- |
|  |  | 1 | 2 | 3 |

| **5.8a** | **Overall, how many different sexual partners did you have during the past 3 months?** |  |  |
| --- | --- | --- | --- |

| **5.8b** | **Have you used a condom with any of your partners in the last 3 months?** | Yes | No |
| --- | --- | --- | --- |
|  |  | 1 | 2 |
|  |  |  | **GO TO NEXT FILTER** |

| **5.8c** | **In the last 3 months when you had sexual intercourse, did the condom ever break/leak/slip off during sex or while pulling out?** | **Yes** | **No** | **Don’t Know** |
| --- | --- | --- | --- | --- |
|  |  | 1 | 2 | 3 |

| **INSTRUCTION** | ***Now I would like to ask you some questions about sexual***  ***health*** |
| --- | --- |

| **INSTRUCTION** | ***The next section deals with infections related to the urinary tracts*** | |
| --- | --- | --- |
| **FEMALES** | | **Q6.8f**  **MALES** |

| **5.8d** | **During the last 12 months have you had an abnormal discharge from your vagina? This may include an unusual smell, colour, or texture.** | Yes | No | Don’t Know |
| --- | --- | --- | --- | --- |
|  |  | 1 | 2 | 3 |

| **5.8e** | **During the last 12 months, have you had an ulcer or sore on or near your vagina?** | Yes | No | Don’t Know |
| --- | --- | --- | --- | --- |
|  |  | 1 | 2 | 3 |

| **INSTRUCTION** | **IF EITHER q5.8d OR q5.8e = YES, THEN GO TO Q5.8h. ELSE GO TO Q5.9** |
| --- | --- |

| **5.8f** | **During the last 12 months, have you had an abnormal discharge from your penis?** | Yes | No | Don’t Know |
| --- | --- | --- | --- | --- |
|  |  | 1 | 2 | 3 |

| **5.8g** | **During the last 12 months, have you had an ulcer or sore on or near your penis?** | Yes | No | Don’t Know |
| --- | --- | --- | --- | --- |
|  |  | 1 | 2 | 3 |

| **5.8h** | **During the last 12 months have you experienced pain when passing urine?** | Yes | No | Don’t Know |
| --- | --- | --- | --- | --- |
|  |  | 1 | 2 | 3 |

| **INSTRUCTION** | **IF EITHER 5.8f OR 5.8g OR 5.8h ‘1’YES, GO TO Q5.8i. OTHERWISE GOT TO Q5.9** |
| --- | --- |

| **5.8i** | **Did you visit a health facility or see a healthcare provider because of these problems?** | Yes | | No |
| --- | --- | --- | --- | --- |
|  |  | 1 | | 2 |
|  | | |  | ***GO TO 5.9*** |

| **5.8j** | **Did you get treatment for these problems from the healthcare provider?** | Yes | No |
| --- | --- | --- | --- |
|  |  | 1 | 2 |

| **INSTRUCTION** | **CHECK 5.2 (NUMBER OF PARTNERS LAST 12-MONTHS):**  **IF LESS THAN 3:**  **INTERVIEWER SAY: *Now I would like to ask you some questions about the ____partners you have had sexual intercourse with in the last 12 months.***  **IF 3 OR GREATER:**  **INTERVIEWER SAY: *Now I would like to ask you some questions about the LAST 3 partners you have had sex with in the past 12 months.***  **INTERVIEWER SAY TO ALL:**  ***Let me assure you again that your answers are completely confidential and will not be told to anyone. If we should come to any question that you don't want to answer, just let me know and we will go to the next question.*** |
| --- | --- |

| **INSTRUCTION** | **IN THE NEXT SECTION, ONLY RECORD UP TO A MAXIMUM OF THREE PERSONS WITH WHOM THE RESPONDENT HAD A SEXUAL RELATIONSHIP WITHIN THE PAST 12 MONTHS** If applicable, this will include their spouse/regular partner and any other persons |
| --- | --- |

|  | | ***Most recent person with whom you had sex*** | ***Second most recent person with whom you had sex*** | ***Third most recent person with whom you had sex*** |
| --- | --- | --- | --- | --- |
| **5.9a** | **What is your relationship?** | Husband / Wife……...1  Live-in partner ……… 2  Girlfriend / Boyfriend  not living with you….... 3  Casual partner ..….…...4  Someone whom you paid for sex …...........…5  Other ...........................6 | Husband / Wife……...1  Live-in partner ……… 2  Girlfriend / Boyfriend  not living with you….... 3  Casual partner ..….…...4  Someone whom you paid for sex …...........…5  Other ...........................6 | Husband / Wife……...1  Live-in partner ……… 2  Girlfriend / Boyfriend  not living with you….... 3  Casual partner ..….…...4  Someone whom you paid for sex …...........…5  Other ...........................6 |
| **5.9b** | **Is your partner a male or a female?** | Male…………..……….1  Female……………..….2 | Male…………..……….1  Female……………..….2 | Male…………..……….1  Female……………..….2 |
| **5.9c** | **What is the highest level of school this partner has completed?** | Primary level……..1  Secondary level….2  Grade 12/ Standard 10/Ntc 3...............3  Further studies incomplete...............4  Diploma/other post school completed...............5  Further degree completed...............6  Don’t know...............98  No schooling..........99 | Primary level……..1  Secondary level….2  Grade 12/ Standard 10/Ntc 3...............3  Further studies incomplete...............4  Diploma/other post school completed...............5  Further degree completed...............6  Don’t know...............98  No schooling..........99 | Primary level……..1  Secondary level….2  Grade 12/ Standard 10/Ntc 3...............3  Further studies incomplete...............4  Diploma/other post school completed...............5  Further degree completed...............6  Don’t know...............98  No schooling..........99 |
| **5.10a** | **What is the employment status of your partner?** | Employed……….…….1  Unemployed……..……2  Student………….…….3  Don’t know................98 | Employed……….…….1  Unemployed……..……2  Student………….…….3  Don’t know................98 | Employed……….…….1  Unemployed……..……2  Student………….…….3  Don’t know................98 |
| **5.10b** | **Where does your partner reside?** | In same area…………..1  In another area…….….2  In same household…..3 | In same area…………..1  In another area…….….2  In same household…..3 | In same area…………..1  In another area…….….2  In same household…..3 |
|  | **Household number of partner, if applicable** | ________ | ________ | ________ |
| **5.10c** | **What is the approximate age of your partner~~?~~** | ________  GO TO 5.11 or 5.12  IF 3 year age gap  Don’t know…………..88  GO TO 5.13 | ________  GO TO 5.11 or 5.12  IF 3 year age gap  Don’t know…………..88  GO TO 5.13 | ________  GO TO 5.11 or 5.12  IF 3 year age gap  Don’t know…………..88  GO TO 5.13 |
| **5.11** | **What is the MOST important reason for having a sexual partner younger than yourself?** | Younger partner is less likely to be infected with STI/HIV………….....…1  Younger partner will give a sexual boost ....2  It is sexually more exciting than having an older or same-age partner……..............…3  Fear of getting old; younger partner rejuvenates..............…4  A younger partner will cure me of HIV/AIDS..5  It is easier to seduce a younger person.......…6  Age is not important…7  Other………......……8 | Younger partner is less likely to be infected with STI/HIV………….....…1  Younger partner will give a sexual boost....2  It is sexually more exciting than having an older or same-age partner……..............…3  Fear of getting old; younger partner rejuvenates ............…4  A younger partner will cure me of HIV/AIDS..5  It is easier to seduce a younger person........6  Age is not important…7  Other………......……8 | Younger partner is less likely to be infected with STI/HIV………….....…1  Younger partner will give a sexual boost ....2  It is sexually more exciting than having an older or same-age partner……..............…3  Fear of getting old; younger partner rejuvenates ...........…4  A younger partner will cure me of HIV/AIDS..5  It is easier to seduce a younger person.......…6  Age is not important…7  Other………......……8 |
| **5.12** | **What is the MOST important reason for having a sexual partner older than yourself?** | Feeling secure...........1  He/she can give financial support .........2  He/she does not cheat...........................3  He/she is experienced and satisfies my sexual needs..........................4  Age is not important...5  Other...........................6 | Feeling secure............1  He/she can give financial support ........2  He/she does not cheat...........................3  He/she is experienced and satisfies my sexual needs..........................4  Age is not important...5  Other...........................6 | Feeling secure............1  He/she can give financial support.........2  He/she does not cheat...........................3  He/she is experienced and satisfies my sexual needs..........................4  Age is not important...5  Other...........................6 |
| **5.13** | **How long ago did you first have sex with your partner?** | A year ago………..1  Below a year……..2  More than a year ago..3  Can’t remember…99 | A year ago………..1  Below a year……..2  More than a year ago..3  Can’t remember…99 | A year ago………..1  Below a year……..2  More than a year ago..3  Can’t remember…99 |
| **5.14** | **When last did you have sex with your partner?** | Months ago [___\|___]  Days ago [___\|___]  Can’t remember…99 | Months ago [___\|___]  Days ago [___\|___]  Can’t remember…99 | Months ago [___\|___]  Days ago [___\|___]  Can’t remember…99 |
| **5.15** | **What type of sex do you have with your partner?** | Yes….No  Vaginal………..1…….2  Anal……………1…….2  Oral sex…….…1…….2 | Yes….No  Vaginal………..1…….2  Anal……………1…….2  Oral sex…….…1…….2 | Yes….No  Vaginal………..1…….2  Anal……………1…….2  Oral sex…….…1…….2 |
| **5.16** | **Are you still sexually active with your partner?** | Yes…………….……1  No…………………...2 | Yes…………….……1  No…………………...2 | Yes…………….……1  No…………………...2 |
| **5.17** | **Do you expect to have sex with your partner again?** | Yes…………….……1  No…………………...2  Do not know………3 | Yes…………….……1  No…………………...2  Do not know………3 | Yes…………….……1  No…………………...2  Do not know………3 |
| **5.18** | **How many times during the last 30 days did you have penetrative sexual intercourse with your partner?** | ________ | ________ | ________ |
| **5.19** | **How often do you use a condom with your partner?** | Every time………….1  Almost every time….2  Sometimes…………3  Never………………..4  GO TO 5.241 | Every time………….1  Almost every time….2  Sometimes…………3  Never………………..4  GO TO 5.241 | Every time……….….1  Almost every time….2  Sometimes…….……3  Never………………..4  GO TO 5.241 |
| **5.20** | **Did you use a condom at last sex?** | Yes……………....…1  No………………......2  GO TO 5.241 | Yes……………....……1  No…………………......2  GO TO 5.241 | Yes……………....……1  No…………………......2  GO TO 6.241 |
| **5.21** | **Was it a male or female condom?** | Male………………….1  Female……………….2 | Male………………….1  Female……………….2 | Male………………….1  Female……………….2 |
| **5.22** | **Who suggested using a condom?** | Yourself…………….1  Your partner………..2  Mutual agreement…3 | Yourself…………….1  Your partner………..2  Mutual agreement…3 | Yourself…………….1  Your partner………..2  Mutual agreement…3 |
| **5.23** | **If you used a condom, what were your reasons for doing so?** | Concern about HIV infection…………....1  People are urged  to use condoms……2  Want to prevent  STI’s ........................3  Want to prevent pregnancy …….…...4  I or partner on ARV…5  Other .......................6  GO TO 5.251 COMPLETED GO TO 6.25 | Concern about HIV infection…………....1  People are urged  to use condoms……2  Want to prevent  STI’s ........................3  Want to prevent pregnancy …….…...4  I or partner on ARV…5  Other .......................6  GO TO 5.251  NCE COMPLETED O TO6.25 | Concern about HIV infection…………....1  People are urged  to use condoms……2  Want to prevent  STI’s ........................3  Want to prevent pregnancy …….…...4  I or partner on ARV…5  Other .......................6  O TO 6.25 |
| **5.24** | **If you did not use a condom, what were your reasons for not doing so?** | Did not have a condom...………….….1  Partner objected.....….2  Used other contraceptive........….. 3  Don’t like them...……..4  Didn’t think it was necessary.............……5  I am married....…….…6  I am faithful /trust them.7  I was drunk/high.....…..8  Other ...........................9 | Did not have a condom...………….….1  Partner objected.....….2  Used other contraceptive........….. 3  Don’t like them...……..4  Didn’t think it was necessary.............……5  I am married....…….…6  I am faithful/trust them 7  I was drunk/high.....…..8  Other ...........................9 | Did not have a condom...………….….1  Partner objected.....….2  Used other contraceptive........….. 3  Don’t like them...……..4  Didn’t think it was necessary.............……5  I am married....…….…6  I am faithful/trust them 7  I was drunk/high.....…..8  Other ...........................9 |
| **5.25** | **The last time you had sex with your partner did you drink alcohol before sex?** | Yes…………….…..1  No………………….2  Can’t remember…..3  (Next partner) | Yes…………….…..1  No………………….2  Can’t remember…..3  (Next partner) | Yes…………….…..1  No………………….2  Can’t remember…..3 |

| **5.26** | **Is it easy to get a condom if you need one? (Male and/female condoms)** | Yes | No | No response |
| --- | --- | --- | --- | --- |
|  |  | 1 | 2 | 3 |

| **INSTRUCTION** | **NON CONDOM USE FILTER (CHECK Q. 5.19)** | |
| --- | --- | --- |
| **SEXUALLY ACTIVE RESPONDENT**  **WHO EVER USED A CONDOM**  ***GOTO 5.27*** | | **SEXUALLY ACTIVE RESPONDENT WHO**  **HAS NEVER USED A CONDOM BEFORE**  ***GO TO SEX FILTER*** |

| **INSTRUCTION** | **DO NOT READ OUT OPTIONS, MULTIPLE RESPONSES POSSIBLE** |
| --- | --- |

| **5.27** | **Where do YOU normally obtain condoms? (Male and/female condoms)** | |
| --- | --- | --- |
| **a** | Government clinic or hospital | 1 |
| **b** | Private clinic or hospital | 2 |
| **c** | Pharmacy/chemist | 3 |
| **d** | Shop/supermarket/café | 4 |
| **e** | Garage/station | 5 |
| **f** | Spaza shop | 6 |
| **g** | Shebeen/tavern/hotel | 7 |
| **h** | Workplace |  |
| **i** | Other | 8 |

| **5.28** | **Did you or your partner pay for the last condom you used or did you get it for free?** (Male and/female condoms) | | |
| --- | --- | --- | --- |
| Paid for | | Free | Not sure/don't know |
| 1 | | 2 | 3 |

| **INSTRUCTION** | **AGE AND SEX FILTER** | |
| --- | --- | --- |
| **MALES** | | **FEMALES**  **8.1** |

| **SECTION 7** | **MALE CIRCUMCISION** |
| --- | --- |

| **INSTRUCTION** | ***I am now going to ask you a few questions on male circumcision.*** |
| --- | --- |

| **7.1** | **Some men are circumcised. Have you been circumcised?** | Yes | No |
| --- | --- | --- | --- |
|  |  | 1 | 2 |
|  |  |  | ***GO TO 7.12*** |

| **INSTRUCTION** | ***I am now going to ask you a sensitive question, please remember that***  ***your responses are confidential*** |
| --- | --- |

| **7.2** | **What type of circumcision did you have?** | |
| --- | --- | --- |
| Partial (where some of the foreskin still remains) | | 1 |
| Full/complete (foreskin is totally removed) | | 2 |
| Don’t know | | 3 |

| **7.3** | **How old were you when you were circumcised?**  **(in years) If at birth - 00** |  |  | Don’t know | 99 |
| --- | --- | --- | --- | --- | --- |

| **7.4** | **Did you receive any explanation or counselling about the circumcision before it was done?** | Yes | No | Too young | Don’t remember |
| --- | --- | --- | --- | --- | --- |
|  |  | 1 | 2 | 3 | 4 |
|  | | | ***GO TO 7.6*** | | |

| **INSTRUCTION** | **DO NOT READ OUT OPTIONS, MULTIPLE RESPONSES POSSIBLE** |
| --- | --- |

| **7.5** | **What were you advised to do after the circumcision about having sex?** | |
| --- | --- | --- |
| **a** | Nothing | 1 |
| **b** | Wait to have sex until the wound is completely healed | 2 |
| **c** | You still have to use a condom even after circumcision | 3 |
| **d** | Have one sex partner | 4 |
| **e** | Other | 5 |

| **7.6** | **Where were you circumcised?** | |
| --- | --- | --- |
| At home | | 1 |
| In hospital/clinic | | 2 |
| In the mountain/in the bush/initiation school | | 3 |
| Circumcision camps | | 4 |
| Mobile clinic circumcision clinic | | 5 |
| Other | | 6 |
| Don’t know | | 7 |

| **7.9** | **What was your main reason for being circumcised?** | |
| --- | --- | --- |
| Traditional practice such as initiation | | 1 |
| Religious reasons | | 2 |
| My parents decided for me | | 3 |
| Health reasons | | 4 |
| Prevent HIV and other STIs | | 5 |
| Other | | 6 |

| **INSTRUCTION** | **ASK MALES WHO ARE NOT CIRCUMCISED** | Yes | No |
| --- | --- | --- | --- |
| **7.12** | **Would you consider being circumcised?** | 1 | 2 |
|  | | ***GO TO 7.14*** |  |

| **7.13** | **Why would you not consider male circumcision?** | |
| --- | --- | --- |
| Personal reasons | | 1 |
| Religious reasons | | 2 |
| Health reasons | | 3 |
| Other | | 4 |

| **SECTION 8** | **HIV COUNSELLING AND TESTING**  ***I am now going you ask you a few questions about HIV testing Please remember that your name is not written anywhere and everything you tell me is confidential.*** |
| --- | --- |

| **8.2** | **Have you ever had an HIV test?** | | Yes | No | No response |
| --- | --- | --- | --- | --- | --- |
|  |  |  | 1 | 2 | 3 |
|  |  | | | ***GO TO 8.24*** | ***GO TO SECTION 9*** |

| **8.3** | **How long ago did you have your most recent HIV test?** | |
| --- | --- | --- |
| 0 to 3 months | | 1 |
| 4 to 6 months | | 2 |
| 7 to 11 months | | 3 |
| Less than a year ago | | 4 |
| Between 1-2 years ago | | 5 |
| Between 2-3 years ago | | 6 |
| Three or more years ago | | 7 |

| **INSTRUCTION** | | ***Please note that you should not tell me about the actual result. I am only interested whether you have been told/informed of the result of the test.*** | | |
| --- | --- | --- | --- | --- |
| **8.4** | **Have you been told/informed of the result of your most recent test*?*** | | Yes | No |
|  |  |  | 1 | 2 |

| **8.6** | **During your most recent HIV test, did you have counselling before the HIV test?** | Yes | No |
| --- | --- | --- | --- |
|  |  | 1 | 2 |

| **8.7** | **During your most recent HIV test, did you have counselling after the HIV test?** | Yes | No |
| --- | --- | --- | --- |
|  |  | 1 | 2 |

| **8.8** | **What was the main reason for going for your last HIV test?** | |
| --- | --- | --- |
| I wanted to know my HIV status | | 1 |
| My partner asked me to go for testing | | 2 |
| I wanted to start a new sexual relationship | | 3 |
| I wanted to get married | | 4 |
| I applied for an insurance policy | | 5 |
| I applied for a loan | | 6 |
| My employer requested it | | 7 |
| I was feeling sick | | 8 |
| I was instructed by a health worker (nurse/doctor) | | 9 |
| I was pregnant | | 10 |
| Workplace campaign | | 11 |
| Other | | 12 |

| **8.9** | **You indicated that you were previously tested for HIV. Are you willing to tell me the last HIV test result you received?** | Yes | No | Never received result |
| --- | --- | --- | --- | --- |
|  |  | 1 | 2 | 3 |
|  |  |  | ***GO TO 8.18*** | |

| **8.10a** | **What was the result of that HIV test?** | Positive | Negative | Indeterminate |
| --- | --- | --- | --- | --- |
|  |  | 1 | 2 | 3 |
|  | | | ***GO TO 8.18*** | |

| **8.10b** | What was the month and year of your first HIV positive test?  **IF “DON’T KNOW” MONTH, THEN RECORD ‘88’**  **IF “DON’T KNOW” YEAR, THEN RECORD ‘8888’** | **MONTH** |  |  |  |  |
| --- | --- | --- | --- | --- | --- | --- |
|  |  | **YEAR** |  |  |  |  |

| **8.11a** | | **Are you currently taking ARVS, that is, antiretroviral medications?** | Yes | | No |
| --- | --- | --- | --- | --- | --- |
|  | |  | 1 | | 2 |
|  |  | | |  | ***GO TO 8.18*** |

| **8.11b** | **Are you taking ARVS, that is, antiretroviral medications, daily?** | Yes | No |
| --- | --- | --- | --- |
|  |  | 1 | 2 |

| **8.12** | **How long have you been taking daily ARVs?**  **RECORD THE ANSWER IN MONTHS IF LESS THAN ONE YEAR. RECORD '00' IF LESS THAN ONE MONTH.** | **Number of years** |  |  |
| --- | --- | --- | --- | --- |
|  |  | **Number of months** |  |  |

| **8.13** | | **Have you ever missed ARV treatment?** | Yes | | No |
| --- | --- | --- | --- | --- | --- |
|  | |  | 1 | | 2 |
|  |  | | |  | ***GO TO 8.18*** |

| **8.14** | **In the past 30 days, have you missed taking any of your ARV pills?** | Yes | No |
| --- | --- | --- | --- |
|  |  | 1 | 2 |

| **INSTRUCTION** | **DO NOT READ OUT OPTIONS, MULTIPLE RESPONSES POSSIBLE** |
| --- | --- |

| **8.15** | **Why did you miss your ARV treatment?** | |
| --- | --- | --- |
| **a** | Forgotten to take ARVs | 1 |
| **b** | I travelled away from my clinic and could not go to another one | 2 |
| **c** | Health reasons | 3 |
| **d** | I had no money to go to the clinic to pick up my treatment | 4 |
| **e** | Health facility had no stock | 5 |
| **f** | Transport problems | 6 |
| **g** | I decided to stop taking treatment because the treatment makes me sick | 7 |
| **h** | Other reasons: Specify | 8 |

| **8.16** | **How long were you not taking the treatment?** | **Days** |  |  |
| --- | --- | --- | --- | --- |
|  |  | **Months** |  |  |

| **INSTRUCTION** | **ONCE YOU HAVE ANSWERED Q8.16 GO TO 8.18** |
| --- | --- |

| **8.17** | **Can you tell me the main reason why you are not taking ARVs daily?** | |
| --- | --- | --- |
| Have trouble taking a tablet everyday/can’t remember | | 1 |
| I don’t think I need it; I don’t feel sick | | 2 |
| I fear people will know that I have HIV if I take it | | 3 |
| Forgotten to take ARVs | | 4 |
| I travelled away from my clinic and could not go to another one | | 5 |
| Health reasons | | 6 |
| I had no money to go to the clinic to pick up my treatment | | 7 |
| Health facility had no stock | | 8 |
| Transport problems | | 9 |
| I decided to stop taking treatment because the treatment makes me sick | | 10 |
| ART makes me fat | | 11 |
| Other reasons: Specify | | 12 |

| **INSTRUCTION** | **SEXUAL ACTIVITY FILTER (CHECK Q6.1)** | |
| --- | --- | --- |
| **HAD SEXUAL PARTNER(S) IN LAST**  **12 MONTHS** | | **NO SEXUAL PARTNERS**  **IN LAST 12 MONTHS NEXT HIV FILTER (q8.24)** |

| **8.18** | **Now that we have discussed your HIV status, I want you to remember main sexual partner in the last 12 months that we had discussed earlier. Did you tell this person the results of your last HIV test?** | Yes | No | Don’t know |
| --- | --- | --- | --- | --- |
|  |  | 1 | 2 | 3 |

| **8.19** | **Have you told other current sexual partners about this test result in the last 12 months?** | Yes | No | No partner |
| --- | --- | --- | --- | --- |
|  |  | 1 | 2 | 3 |

| **8.20** | **In the past six months, how many sex partners have you had**  **whose HIV status you did not know at the time that you had sex?** |  |  |
| --- | --- | --- | --- |

| **8.21** | **In the past six months, how many sex partners have you had who**  **did not know your HIV status when you had sex with them?** |  |  |
| --- | --- | --- | --- |

| **8.22** | **Have you ever taken an HIV test with any of your sex partners where you both received the test results together?** | Yes | No |
| --- | --- | --- | --- |
|  |  | 1 | 2 |
|  | | ***GO TO SECTION 10*** |  |

| **8.23** | **What was the main reason why have not you tested for HIV as a couple?** | |
| --- | --- | --- |
| Never discussed it | | 1 |
| Discussed it, but decided not to do it | | 2 |
| My partner refused to allow me to go for a test | | 3 |
| I did not want to be tested although my partner wanted to do so | | 4 |
| My partner and I already know our status | | 5 |
| Don’t know where to get couples testing | | 6 |
| Other (Specify) | | 7 |

| **INSTRUCTION** | **HIV TESTING FILTER** | |
| --- | --- | --- |
| **NEVER HAD AN HIV TEST carry on to 8.24** | | **HAD AN HIV TEST GO TO SECTION 9** |

| **INSTRUCTION** | | **DO NOT READ OUT OPTIONS, MULTIPLE RESPONSES POSSIBLE** | | | |
| --- | --- | --- | --- | --- | --- |
| **8.24** | | **What were your reasons for not going for an HIV test?** | | | |
| **a** | Do not know where to get tested | | | | 1 |
| **b** | Do not think that I have HIV | | | | 2 |
| **c** | Not at risk for HIV | | | | 3 |
| **d** | Trust partner | | | | 4 |
| **e** | Afraid to find out that I might be HIV positive | | | | 5 |
| **f** | Not ready to have an HIV test | | | | 6 |
| **g** | Concerned about CONFIDENTIALITY | | | | 7 |
| **h** | Concerned about STIGMA, DISCRIMINATION, or REJECTION | | | | 8 |
| **i** | Concerned about LOSING MY JOB | | | | 9 |
| **j** | Concerned about the STANDARD OF SERVICE | | | | 10 |
| **k** | Haven’t got around to do it | | | | 11 |
| **l** | Other: specify | | | | 12 |
| **INSTRUCTION** | | | **HIV POSITIVE** | | |
| **IF HIV STATUS UNKNOWN OR NEGATIVE** | | | | **HIV POSITIVE GO TO SECTION 11** | |

| **SECTION 9** | **HIV RISK PERCEPTION** |
| --- | --- |

| **INSTRUCTION** | ***I am now going to ask you some questions on how you perceive your risk to HIV infection Please remember that your name is not written anywhere and everything you tell me is confidential.*** |
| --- | --- |

| **INSTRUCTION** | **READ EACH STATEMENT** |
| --- | --- |

| **9.1** | **On a scale of 1 to 4 (with 1 being low and 4 being high), how would you rate yourself in terms of risk of becoming infected with HIV?** | | |
| --- | --- | --- | --- |
| You are definitely going to get infected with HIV | | **Go to** **9.3** | 4 |
| You are probably going to get infected | | **Go to** **9.3** | 3 |
| You probably won’t get infected | | | 2 |
| You definitely will not get infected with HIV | | | 1 |
| Already HIV positive | | **Go to** **10.1** | 9 |

| **INSTRUCTION** | | **DO NOT READ OUT OPTIONS, MULTIPLE RESPONSES POSSIBLE** | |
| --- | --- | --- | --- |
| **9.2** | **What are your reasons for believing so*?*** | | |
| **a** | Never had sex before | | 1 |
| **b** | Abstain from sex | | 2 |
| **c** | Faithful to his/her partner | | 3 |
| **d** | Trust his/her partner | | 4 |
| **e** | Use condoms | | 5 |
| **f** | Know his/her HIV status | | 6 |
| **g** | Know the HIV status of his/her partner | | 7 |
| **h** | Do not have sex with sex workers/prostitutes | | 8 |
| **i** | Protected by ancestors | | 9 |
| **j** | Protected by God | | 10 |
| **k** | I am not at risk for HIV | | 11 |
| **l** | Other | | 12 |

| **INSTRUCTION** | **ONCE YOU HAVE ASKED QUESTION 9.2 SKIP TO Q9.4** |
| --- | --- |

| **INSTRUCTION** | | **DO NOT READ OUT OPTIONS, MULTIPLE RESPONSES POSSIBLE** | |
| --- | --- | --- | --- |
| **9.3** | **What are your reasons for believing so*?*** *(Respondents who answered 3 or 4 in Q9****.1)*** | | |
| **a** | Sexually active | | 1 |
| **b** | Had many sexual partners | | 2 |
| **c** | Don't use condoms | | 3 |
| **d** | Don't always use condoms | | 4 |
| **e** | Don't trust his/her partner | | 5 |
| **f** | I am sick | | 6 |
| **g** | My partner is sick | | 7 |
| **h** | My partner died of AIDS | | 8 |
| **i** | Had an accident/cuts | | 9 |
| **j** | I am HIV positive | | 10 |
| **k** | Other | | 11 |

| **9.4** | **Scientists are now studying a medication where, if taken orally every day, can reduce a person’s chances of getting HIV infection. If such a medication was available, would you want to take it?** | Yes | No | Don’t know |
| --- | --- | --- | --- | --- |
|  |  | 1 | 2 | 3 |

| **9.5** | **An HIV self-test kit is a method where people can**  **test for HIV in private or at home. If such a kit**  **was available to you, would you be willing to use**  **it to test yourself?** | Yes | No | Don’t know |
| --- | --- | --- | --- | --- |
|  |  | 1 | 2 | 3 |

| **9.6** | **Have you heard about drug treatments that can help reduce the risk of HIV infection if a person has been raped?** | Yes | No |
| --- | --- | --- | --- |
|  |  | 1 | 2 |

| **SECTION 10** | **ALCOHOL USE** |
| --- | --- |

| **INSTRUCTION** | ***The next section contains questions on the use of alcohol*** |
| --- | --- |
|  | **USE THE EXAMPLE BELOW TO HELP YOU UNDERSTAND WHAT A STANDARD UNIT OR A STANDARD DRINK IS:** |

| One standard drink:   \| 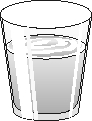 \| *A single tot of spirits*  *(e.g., 25ml at 43%)* \| 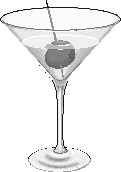 \| *A small glass of liqueur or aperitif*  *(e.g,. 25ml at 30%)* \| \| \| \| --- \| --- \| --- \| --- \| --- \| --- \| \| 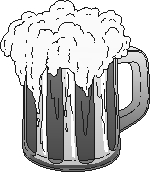 \| *1 can of ordinary beer*  *(e.g., 340ml at 5%)* \| 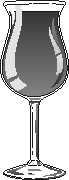 \| *1 glass of wine*  *(e.g., 120ml at 12%)* \| 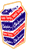 \| *Carton of ordinary commercial sorghum beer (e.g., 500ml at 3%)* \| |
| --- | --- | --- | --- | --- | --- | --- | --- | --- | --- | --- | --- | --- |

| **10.1** | **Have you ever had a drink containing alcohol?** | Yes | No |
| --- | --- | --- | --- |
|  |  | 1 | 2 |
|  |  |  | ***GO TO 11.1*** |

| **10.2** | **How often did you have a drink containing alcohol in the past 12 months?** | | | | |
| --- | --- | --- | --- | --- | --- |
|  | Not in the past 12 months | Once a month or less | 2-4 times a month | 2-3 times a week | 4 or more times a week |
|  | 1 | 2 | 3 | 4 | 5 |
|  | ***GO TO 10.5*** |  | | | |

| **10.3** | **How many drinks containing alcohol do you have on a typical day when you are drinking?** | | | | |
| --- | --- | --- | --- | --- | --- |
|  | 1 or 2 | 3 or 4 | 5 or 6 | 7 to 9 | 10 or more |
|  | 1 | 2 | 3 | 4 | 5 |

| **INSTRUCTION** | | **READ EACH QUESTION** | **Never** | **Less than monthly** | **Monthly** | **Weekly** | **Daily or almost daily** |
| --- | --- | --- | --- | --- | --- | --- | --- |
| **10.4** | |  |  |  |  |  |  |
| **a** | How often do you have (***for men***) five or more and (***for women***) four or more drinks on one occasion? | | 1 | 2 | 3 | 4 | 5 |
| **b** | How often during the past 12 months were you not able to stop drinking once you had started? | | 1 | 2 | 3 | 4 | 5 |
| **c** | How often during the past 12 months did you fail to do what was normally expected of you because of drinking? | | 1 | 2 | 3 | 4 | 5 |
| **d** | How often during the past 12 months did you need a drink first thing in the morning to get yourself going after a heavy drinking session? | | 1 | 2 | 3 | 4 | 5 |
| **e** | How often during the past 12 months did you feel guilt or remorse after drinking? | | 1 | 2 | 3 | 4 | 5 |
| **f** | How often during the past 12 months were you unable to remember what happened the night before because of your drinking? | | 1 | 2 | 3 | 4 | 5 |

| **10.5** | **Have you or someone else been injured as a result of your drinking?** | | |
| --- | --- | --- | --- |
| No | | **Yes,** but not in the past 12 months | **Yes**, during the past 12 months |
| 1 | | 2 | 3 |

| **10.6** | **As a result of your drinking, have you and others been involved in violent actions and aggression?** | | |
| --- | --- | --- | --- |
| No | | **Yes,** but not in the past 12 months | **Yes**, during the past 12 months |
| 1 | | 2 | 3 |

| **10.7** | **Has a concerned relative, friend, doctor, or other health worker ever suggested that you should cut down on your drinking?** | | |
| --- | --- | --- | --- |
| No | | **Yes,** but not in the past 12 months | **Yes**, during the past 12 months |
| 1 | | 2 | 3 |

| **SECTION 11** | **USE OF OTHER SUBSTANCES** |
| --- | --- |

| **INSTRUCTION** | **The next section deals with the use of drugs. I once again want to assure you that the information you give us is combined with all the respondents and we do not analyse the information of one person. I once again want to confirm my earlier guarantee of confidentiality. As interviewer, I also had to sign an undertaking never to speak to others about the interviews I conduct.** |
| --- | --- |

| **11.1** | **Besides drugs prescribed by a health professional, have you ever used a drug by injection?** | | |
| --- | --- | --- | --- |
| **No**, never | | **Yes,** in the past 3 months | **Yes**, but not in the past 3 months |
| 1 | | 2 | 3 |
| ***GO TO 12.1*** | |  |  |

| **11.2** | **Have you ever shared injection needles?** | | |
| --- | --- | --- | --- |
| **No**, never | | **Yes,** in the past 3 months | **Yes**, but not in the past 3 months |
| 1 | | 2 | 3 |

| **SECTION 12** | **HEALTH QUESTIONS** |
| --- | --- |

| **INSTRUCTION** | ***The next section deals with some questions pertaining to your own health as well as services you received in clinics/hospitals or elsewhere. Please remember that your name is not written anywhere and everything you tell me is confidential.*** |
| --- | --- |

| **12.1** | **In general, would you say that your health is excellent, good, fair, or poor?** | |
| --- | --- | --- |
| Excellent | | 1 |
| Good | | 2 |
| Fair | | 3 |
| Poor | | 4 |

| **12.2** | **When was the last time you went to see a health professional (doctor, nurse, traditional healer, etc.)?** | |
| --- | --- | --- |
| Within the past six months | | 1 |
| More than six months but not more than a year ago | | 2 |
| More than one year ago | | 3 |
| Never | | 4 |

| **12.3** | **Where do you usually obtain health care?** | |
| --- | --- | --- |
| Government hospital | | 1 |
| Day hospital/clinic/community health Centre | | 2 |
| Mobile clinic | | 3 |
| Family planning clinic | | 4 |
| Private hospital/clinic | | 5 |
| Pharmacy | | 6 |
| Private doctor | | 7 |
| Other private medical | | 8 |
| Other | | 9 |

| **12.4** | **In the past 12 months, have you been hospitalised for any illness?** | Yes | No |
| --- | --- | --- | --- |
|  |  | 1 | 2 |
|  |  |  | ***GO TO 12.7*** |

| **12.5** | **How many times have you been admitted to hospital during the past 12 months?** |  |  |
| --- | --- | --- | --- |

| **12.6** | **What was the total time you spent in hospital during the past 12 months? (In days)** |  |  |  |
| --- | --- | --- | --- | --- |

| **INSTRUCTION** | **ONLY ASKED (ii) FOR EACH ILLNESS IF YES IN (i)** |
| --- | --- |

| **12.7** | Have you been diagnosed with any of the following illnesses? | **i. Diagnosed with illness** | | | **ii. Are you currently taking medicines for this disease?** | |
| --- | --- | --- | --- | --- | --- | --- |
|  |  | **Yes** | **No** | **Refused** | **Yes** | **No** |
| **a** | Hypertension/high blood pressure | 1 | 2 | 3 | 1 | 2 |
| **b** | Diabetes | 1 | 2 | 3 | 1 | 2 |
| **c** | Cancer | 1 | 2 | 3 | 1 | 2 |
| **d** | Heart disease | 1 | 2 | 3 | 1 | 2 |
| **e** | Silicosis (dust disease) | 1 | 2 | 3 | 1 | 2 |

| **12.8** | **Do you currently smoke tobacco products?** | Yes | No |
| --- | --- | --- | --- |
|  |  | 1 | 2 |
|  |  |  | ***GO TO 12.11*** |

| **12.9** | **How often do you smoke these products?** | |
| --- | --- | --- |
| Daily | | 1 |
| Less than daily | | 2 |
| Don’t know/unknown | | 3 |

| **12.10** | **On average how much do you smoke per week?** |  |  |
| --- | --- | --- | --- |
| **a** | Number of manufactured cigarettes |  | per week |
| **b** | Number of hand rolled cigarettes |  | per week |
| **c** | Number of tobacco pipes |  | per week |
| **d** | unknown | 999 | per week |

| **12.11** | **Are you covered by a Medical Aid or Medical Benefit Scheme?** | Yes | No |
| --- | --- | --- | --- |
|  |  | 1 | 2 |

| **SECTION 15** | **HOUSEHOLD RELATIONS** |
| --- | --- |

| **INSTRUCTION** | ***I am now going to ask you questions about relationships.*** |
| --- | --- |

| **15.0** | ***Have you ever been in a relationship?*** | Yes | No |
| --- | --- | --- | --- |
|  |  | 1 | 2 |
|  | | | ***END*** |

| **INSTRUCTION** | **ONLY ADMINISTERED TO ONE HOUSEHOLD MEMBER** |
| --- | --- |
|  | ***READ TO THE RESPONDENT***  ***I am now going to ask you questions about relationships. You may find some of these questions very personal.***  ***Let me assure you that your answers are completely confidential and will not be told to anyone and no one else in your household will know that you were asked these questions.*** |

| **13.1** | **Did your partner ever do any of the following things to you that could hurt you?** | Yes | No |
| --- | --- | --- | --- |
| **a** | Push you, shake you, or throw something at you? | 1 | 2 |
| **b** | Slap you? | 1 | 2 |
| **c** | Twist your arm or pull your hair? | 1 | 2 |
| **d** | Punch you with his fist or with something | 1 | 2 |
| **e** | Kick you, drag you, or beat you up? | 1 | 2 |
| **f** | Try to choke you or burn you on purpose? | 1 | 2 |
| **g** | Threaten or attack you with a knife, gun, or other weapon? | 1 | 2 |
| **h** | Physically force you to have sexual intercourse with him/her when you did not want to | 1 | 2 |
| **i** | Physically force you to perform any other sexual acts you did not want to | 1 | 2 |
| **j** | Force you with threats or in any other way | 1 | 2 |
| **k** | Perform sexual acts you did not want to? | 1 | 2 |

| **13.2** | **Did the following ever happen as a result of what your partner did to you?** | Yes | No |
| --- | --- | --- | --- |
| **a** | You had cuts, bruises, or aches? | 1 | 2 |
| **b** | You had eye injuries, sprains, dislocations, or burns? | 1 | 2 |
| **c** | You had deep wounds, broken bones, broken teeth? | 1 | 2 |
| **d** | Other serious injury? | 1 | 2 |

| **13.3** | **In the last 12 months, how often has your partner physically hurt you?** | |
| --- | --- | --- |
| **a** | Often, | 1 |
| **b** | Only sometimes, | 2 |
| **c** | Not at all? | 3 |

| **13.4** | **Have you ever hit, slapped, kicked, or done anything else to physically hurt your partner?** | |
| --- | --- | --- |
| **a** | Yes | 1 |
| **b** | No | 2 |

**Exit questions**

|  | | | |
| --- | --- | --- | --- |
| **14.1** | Thank you, we are almost finished. Was it difficult or easy to do this interview on the computer | **Easy** | **Difficult** |
|  |  | 1 | 2 |

|  | | | | |
| --- | --- | --- | --- | --- |
| **14.2** | Do you prefer such an interview with a person or with a computer? | Prefer a person | Prefer a computer | Both are OK |
|  |  | 1 | 2 | 3 |

|  | | | | |
| --- | --- | --- | --- | --- |
| **14.3** | Are you more likely to tell private things about yourself to a person or a computer? | Prefer a person | Prefer a computer | Both are OK |
|  |  | 1 | 2 | 3 |

|  |  | | | | | |
| --- | --- | --- | --- | --- | --- | --- |
| **14.4** | What was the main reason for agreeing to take part in this survey? *(Read categories, check all that apply)* | TB screening | HIV testing | Other testing | Reimbursement | **All mentioned** |
|  |  | 1 | 2 | 3 | 4 | 5 |

If there is another reason not mentioned, please tell me( 6) ……………………………………………

***THANK YOU VERY MUCH FOR AGREEING TO TAKE PART AND ASSIST US IN THIS IMPORTANT RESEARCH PROJECT.***

| **INTERVIEW ENDING TIME:** |  |  | : |  |  |
| --- | --- | --- | --- | --- | --- |

| **INSTRUCTION** | WHAT IS THE PERSON NUMBER OF THE PARTNER OF THIS PERSON? (Get the person number from the VP questionnaire)? |  |  |
| --- | --- | --- | --- |
| **14.1** |  |  |  |

### **Appendix 6: Individual Questionnaire for children aged 12 to 14 years**

***Joint Tuberculosis and HIV Pilot Survey***

Questionnaire number:

Barcode

**JOINT TUBERCULOSIS AND HIV SURVEY PILOT 2019**

| **A** | **GEOGRAPHIC AND INTERVIEW PARTICULARS** | | | | | | | | |
| --- | --- | --- | --- | --- | --- | --- | --- | --- | --- |
| Province | | | | | | | | |  |
| Cluster number | | | | |  |  |  |  |  |
| Small area layer | |  |  |  |  |  |  |  |  |
| Visiting point number (from the map) | | | | |  |  |  |  |  |
| Person number of respondent | | | | | | | |  |  |

| **B** | **INTERVIEW DETAILS** | | | | | | | | | |
| --- | --- | --- | --- | --- | --- | --- | --- | --- | --- | --- |
|  | | Year | | Month | | Day | | Time code | Response code |  |
| First visit | | 1 |  |  |  |  |  |  |  |  |
| Second visit | | 1 |  |  |  |  |  |  |  |  |
| Third visit | | 1 |  |  |  |  |  |  |  |  |
| Final response code | | | | | | | | |  |  |
| **Time code**  1 = Morning till 12:00  2 = 12:00-16:00  3 = 16:00-18:00  4 = 18:00-20:00  5 = 20:00 and later | | **Response code**  1 = Interview completed and sample taken  2 = Interview completed but sample not taken  3 = Appointment made for interview and/or sample  4 = Selected respondent not at home  5 = Refusal by head of household  6 = Refusal by respondent  7 = Other | | | | | | | |  |

| **INTERVIEW STARTING TIME:** |  |  | : |  |  |
| --- | --- | --- | --- | --- | --- |

| **INTERVIEWER: NAME AND NUMBER:………………………………………..** |  |  |  |  |
| --- | --- | --- | --- | --- |

| **C** | **REFUSAL PARTICULARS (IF APPLICABLE)** | |
| --- | --- | --- |
| At what point did the respondent refuse?  SPECIFY | |  |

1 = At the reception desk

2 = After explanation of the survey and the process ( group information session)

4 = During the individual interview

5 = After the individual interview when requested to do the test

6 = Other

Refusals during individual interview

20 = Objected to providing any/some information on the topic

21 = Objected to providing personal/confidential information

22 = Unable to provide requested information

23 = Refused to continue because he/she got irritated/bored

24 = Refused to continue because he/she got angry

25 = Refused to continue because he/she lost interest or got tired

26 = Refused to continue because he/she was in a hurry

27 = Other

Refusal to provide a blood sample

40 = Apprehensive of blood sample being taken

41 = Against religious beliefs to provide a blood sample

42 = Did not want to know HIV status

43 = Fear a breach of confidentiality

44 = Did not trust the interviewers

45 = Recently had an HIV test

46 = Did not to disclose status to parents/guardian

47 = Other

| **Collection and use of demographic information after refusal to participate or withdrawal from the study** | | |
| --- | --- | --- |
|  | I agree that you can record and use information on my age, sex, and level of education. | Child  Yes………………………………………..1  No.………………………………………..2  Parent/ guardian  Yes………………………………………..1  No.………………………………………..2 |

| **GENERAL INSTRUCTION** | **CIRCLE THE CODE NEXT TO THE APPROPRIATE ANSWER.**  **IF INDICATED READ THE ANSWER OPTIONS. *Please remember that your name is not written anywhere and everything you tell me is confidential.*** |
| --- | --- |

| **SECTION 1** | **RESPONDENT’S BIOGRAPHICAL DATA** |
| --- | --- |

| **1.1a** | **How old were you at your last birthday? (*Age of the respondent*)** |  |  |
| --- | --- | --- | --- |

| **1.1b** | **What your date of birth?** | | | | | |  |
| --- | --- | --- | --- | --- | --- | --- | --- |
| Year | | | | Month | |  | |
|  | | |  |  |  |  |  |

| **INSTRUCTION** | | **DO NOT ASK. RECORD SEX** | Male | Female |
| --- | --- | --- | --- | --- |
| **1.2** | **Sex of the respondent** | | 1 | 2 |

| **INSTRUCTION** | | **DO NOT ASK. RECORD RACE** | | | |
| --- | --- | --- | --- | --- | --- |
| **1.3** | | **Race of the respondent** | | | |
| African | White | | Coloured | Indian/Asian | Other |
| 1 | 2 | | 3 | 4 | 5 |

| **1.4** | **What is your nationality?** | |
| --- | --- | --- |
| South African citizen | | 1 |
| Non-citizen (permanent resident ) | | 2 |
| Non-citizen (Refugee) | | 3 |
| Other | | 4 |

| **1.5** | **Are you currently attending school?** | |
| --- | --- | --- |
| Yes | | 1 |
| No (Left school) ***GO TO 1.7*** | | 2 |

| **1.6** | **What grade are you attending this year?** | |
| --- | --- | --- |
| Grade 3/Standard 1/Abet 1 | | 1 |
| Grade 4 /Standard 2/Abet 2 | | 2 |
| Grade 5 /Standard 3/Abet 2 | | 3 |
| Grade 6 /Standard 4/Abet 3 | | 4 |
| Grade 7/Standard 5/Abet 3 | | 5 |
| Grade 8 /Standard 6/Abet 3 | | 6 |
| Grade 9 /Standard 7/Abet 3 | | 7 |
| Grade 10/Standard 8/Ntc 1 | | 8 |
| ***GO TO Q1.9*** |  |  |

| **1.7** | **If ‘No’, have you completed a grade successfully at school?** | |
| --- | --- | --- |
| Grade 2/Sub b/Class 2 | | 1 |
| Grade 3/Standard 1/Abet 1 | | 2 |
| Grade 4 /Standard 2/Abet 2 | | 3 |
| Grade 5 /Standard 3/Abet 2 | | 4 |
| Grade 6 /Standard 4/Abet 3 | | 5 |
| Grade 7/Standard 5/Abet 3 | | 6 |
| Grade 8 /Standard 6/Abet 3 | | 7 |
| Grade 9 /Standard 7/Abet 3 | | 8 |
| No schooling | | 9 |

| **INSTRUCTION** | | **DO NOT READ OUT OPTIONS. MULTIPLE RESPONSES POSSIBLE** | | | |
| --- | --- | --- | --- | --- | --- |
| **1.9** | **Is your biological mother alive?** | | Yes | No | Don’t know |
|  |  |  | 1 | 2 | 3 |
|  | | | | ***GO TO 1.11*** | ***GO TO 1.12*** |

| **1.10** | **Does your biological mother live in this household?** | Yes | No |
| --- | --- | --- | --- |
|  |  | 1 | 2 |
|  |  | ***GO TO 1.12*** | |

| **1.11** | **How old were you when she passed away? (*Age in years*)** |  |  | Don’t Know |
| --- | --- | --- | --- | --- |

| **1.12** | | **Is your biological father alive?** | Yes | No | | Don’t know | | |
| --- | --- | --- | --- | --- | --- | --- | --- | --- |
|  |  |  | 1 | 2 | | 3 | | |
|  | | | | ***GO TO 1.14*** | | ***GO TO 2.1*** | | |
| **1.13** | **Does your biological father live in this household?** | | | | Yes | | No |  |
|  |  |  |  |  | 1 | | 2 |  |
|  |  |  |  |  | ***GO TO SECTION 2*** | | |  |

| **1.14** | **How old were you when he passed away? (*Age in years*)** |  |  | Don’t Know |
| --- | --- | --- | --- | --- |

| **SECTION 2** | **HIV AND AIDS KNOWLEDGE, PERCEPTIONS, AND STIGMA. *Please remember that your name is not written anywhere and everything you tell me is confidential.*** |
| --- | --- |

| **INSTRUCTION**  **2.1** | | ***I am now going to ask you a number of questions about knowledge and perceptions of HIV and AIDS*** | Yes | No | Don’t know |
| --- | --- | --- | --- | --- | --- |
| **a** | Can AIDS be cured? | | 1 | 2 | 3 |
| **b** | Can a person reduce the risk of HIV by having fewer sexual partners? | | 1 | 2 | 3 |
| **c** | Can a healthy-looking person have HIV? | | 1 | 2 | 3 |
| **d** | Can HIV be transmitted from a mother to her unborn baby? | | 1 | 2 | 3 |
| **e** | Can the risk of HIV transmission be reduced by having sex with only one uninfected partner who has no other partners? | | 1 | 2 | 3 |
| **f** | Can a person get HIV by sharing food with someone who is infected? | | 1 | 2 | 3 |
| **g** | Can a person reduce the risk of getting HIV by using a condom every time he/she has sex? | | 1 | 2 | 3 |
| **h** | Can medical male circumcision reduce the risk of HIV infection in males? | | 1 | 2 | 3 |
| **i** | Are there medicines that people with HIV or AIDS can take to help them live longer? | | 1 | 2 | 3 |

| **INSTRUCTION** | | | ***Now I want to ask you some questions relating to people living with HIV/AIDS*** | **Yes** | **No** | **Not sure** |
| --- | --- | --- | --- | --- | --- | --- |
| **2.2** | |  |  |  |  |  |
| **a** | If you knew that a shopkeeper or food seller had HIV, would you buy food from them? | | | 1 | 2 | 3 |
| **b** | Would you be willing to care for a family member with AIDS? | | | 1 | 2 | 3 |
| **c** | If a teacher has HIV but is not sick, he or she should be allowed to continue teaching | | | 1 | 2 | 3 |
| **d** | Would you be willing to share food with someone who has HIV or AIDS? | | | 1 | 2 | 3 |
| **e** | Would you want to keep the HIV-positive status of a family member a secret? | | | 1 | 2 | 3 |
| **f** | Are you comfortable talking to at least one member of your family about HIV/AIDS? | | | 1 | 2 | 3 |
| **g** | Would you play with someone who has HIV or AIDS? | | | 1 | 2 | 3 |

| **SECTION 3** | **SEXUAL DEBUT.** |
| --- | --- |

| **INSTRUCTION** | ***I now have to ask you very sensitive questions on sex and other sex-related matters. Please remember that your name will not be recorded anywhere in this questionnaire and the information you give will be kept confidential.*** |
| --- | --- |

| **3.1** | **Have you ever had sexual intercourse? [***That is when the penis is in the vagina or anu***s]** | Yes | No | No response |
| --- | --- | --- | --- | --- |
|  |  | 1 | 2 | 3 |
|  | | | ***GO TO Q6.1*** | |

| **3.2** | **How old were you when you had sex for the first time?** |  |  |
| --- | --- | --- | --- |

| **SECTION 4** | **KNOWLEDGE AND SUPPORT *Please remember that your name is not written anywhere and everything you tell me is confidential.*** |
| --- | --- |

| **INSTRUCTION** | **DO NOT READ OUT OPTIONS. MULTIPLE RESPONSES POSSIBLE** |
| --- | --- |

| **4.1** | **Has a parent/guardian ever talked to you about sex?** | Yes | No | No response |
| --- | --- | --- | --- | --- |
|  |  | 1 | 2 | 3 |

| **4.2** | **Has a parent/guardian ever talked to you about sexual abuse?** | Yes | No | No response |
| --- | --- | --- | --- | --- |
|  |  | 1 | 2 | 3 |

| **4.3** | **Have you ever discussed HIV or AIDS with your parent/guardian?** | Yes | No | No response |
| --- | --- | --- | --- | --- |
|  |  | 1 | 2 | 3 |

| **INSTRUCTION** | ***Please tell me whether you agree or disagree with the following statements?*** |
| --- | --- |

| **4.4** | | It is good for a parent to talk to their child about: | **Agree** | **Disagree** | **Don’t know** |
| --- | --- | --- | --- | --- | --- |
| **a** | Sexual abuse | | 1 | 2 | 3 |
| **b** | HIV and AIDS | | 1 | 2 | 3 |
| **c** | Relationships | | 1 | 2 | 3 |

| **INSTRUCTION** | | **DO NOT READ OUT OPTIONS. MULTIPLE RESPONSES POSSIBLE** | |
| --- | --- | --- | --- |
| **4.5** | **What should you do to protect yourself against sexual abuse?** | | |
| **a** | Should not accept gifts or money from a stranger | | 1 |
| **b** | Should not talk to a stranger | | 2 |
| **c** | Should not accept a lift from a stranger | | 3 |
| **d** | Should not accompany a stranger if requested | | 4 |
| **e** | Should tell an adult if someone tries to touch my private parts | | 5 |
| **f** | Should tell an adult if someone makes sexual suggestions to me | | 6 |
| **g** | Should not let strangers into the house | | 7 |
| **h** | Should not leave the yard/property without telling an adult | | 8 |
| **i** | Should make sure there is always an adult nearby who will help me | | 9 |
| **j** | Should not go out of the home alone | | 10 |
| **k** | Know the childline number (add number) | | 11 |
| **l** | Other | | 12 |

| **SECTION 5** | **HEALTH QUESTIONS.** |
| --- | --- |

| **INSTRUCTION** | ***The next section deals with services you received in clinics/hospitals or elsewhere, as well as some questions pertaining to your own health. Please remember that your name is not written anywhere and everything you tell me is confidential.*** |
| --- | --- |

| **5.1** | **In general, would you say that your health is excellent, good, fair or poor?** | |
| --- | --- | --- |
| Excellent | | 1 |
| Good | | 2 |
| Fair | | 3 |
| Poor | | 4 |

| **5.2** | **When was the last time you went to see a health personnel (doctor, nurse, traditional healer, etc.)?** | |
| --- | --- | --- |
| Within the past six months | | 1 |
| More than six months, but not more than a year ago | | 2 |
| One year ago or longer | | 3 |
| Never | | 4 |

| **5.3** | **Where do you usually obtain health care?** | |
| --- | --- | --- |
| Government hospital | | 1 |
| Day hospital/clinic community health centre | | 2 |
| Mobile clinic | | 3 |
| Private hospital/clinic | | 4 |
| Other private medical | | 5 |
| Other | | 6 |

| **5.4** | **In the last 12 months, have you been hospitalised for any illness?** | Yes | No |
| --- | --- | --- | --- |
|  |  | 1 | 2 |
|  |  |  | ***GO TO SEX Filter (Q6)*** |

| **5.5** | **How many times have you been admitted to hospital during the past 12 months?** |  |  |
| --- | --- | --- | --- |

| **5.6** | **What was the total time you spent in hospital during the last 12 months? (In days)** |  |  |  |
| --- | --- | --- | --- | --- |

| **MALE CHILD** | **Q7.1**  **FEMALE CHILD** |
| --- | --- |

| **SECTION 6** | **MALE CIRCUMCISION.** |
| --- | --- |

| **INSTRUCTION** | ***I am now going to ask you questions on male circumcision. Please remember that your name is not written anywhere and everything you tell me is confidential.*** |
| --- | --- |

| **6.1** | **Have you been circumcised?** | Yes | No |
| --- | --- | --- | --- |
|  |  | 1 | 2 |
|  |  |  | ***GO TO 6.7*** |

| **INSTRUCTION** | ***I am now going to ask you a sensitive question, please remember that***  ***your responses are confidential*** |
| --- | --- |

| **6.1a** | **What type of circumcision did you have?** | |
| --- | --- | --- |
| Partial (some foreskin remains) | | 1 |
| Full/complete (foreskin is totally removed) | | 2 |
| Don’t know | | 3 |

| **6.2** | **How old were you when you were circumcised? (in years)**  **If at birth – 00** |  |  |
| --- | --- | --- | --- |

| **6.3** | **Where were you circumcised?** | |
| --- | --- | --- |
| At home | | 1 |
| In hospital/clinic | | 2 |
| In the mountains/ in the bush/initiation school | | 3 |
| Other | | 4 |
| Circumcision camps | | 5 |
| Mobile circumcision clinics | | 6 |
| Don’t know | | 7 |

| **6.4** | **Who performed the circumcision?** | |
| --- | --- | --- |
| Doctor | | 1 |
| Spiritual or religious leader | | 2 |
| Traditional circumciser | | 3 |
| Other | | 4 |
| Don’t know | | 5 |

| **6.5** | **What was your main reason for being circumcised?** | |
| --- | --- | --- |
| Traditional practice such as initiation | | 1 |
| Religious reasons | | 2 |
| My parents decided for me | | 3 |
| Health reasons | | 4 |
| Prevent HIV and other STIs | | 5 |
| Other | | 6 |

| **6.6** | **Did you experience complications following circumcision?** | Yes | No | Don’t know |
| --- | --- | --- | --- | --- |
|  |  | 1 | 2 | 3 |
|  | | ***GO TO Q7.1*** | ***GO TO Q7.1*** | ***GO TO Q7.1*** |

| **6.7** | **Would you consider being circumcised?** | Yes | No |
| --- | --- | --- | --- |
|  |  | 1 | 2 |

| **SECTION 7** | **HIV RISK PERCEPTION AND TESTING. *Please remember that your name is not written anywhere and everything you tell me is confidential.*** |
| --- | --- |

| **7.1** | **Do you think you have a chance of getting HIV?** | | | |
| --- | --- | --- | --- | --- |
| Yes | | No | Don’t know | HIV infected |
| 1 | | 2 | 3 | 4 |
|  | | ***GO TO 7.3*** | ***GO TO 7.3*** | ***GO TO 7.4*** |

| **INSTRUCTION** | | **DO NOT READ OUT OPTIONS. MULTIPLE RESPONSES POSSIBLE** | |
| --- | --- | --- | --- |
| **7.2** | **What are your reasons for believing so*?*** | | |
| **a** | I am sexually active | | 1 |
| **b** | I was born with HIV | | 2 |
| **c** | I have had many sexual partners | | 3 |
| **d** | I don't use condoms | | 4 |
| **e** | I don't always use condoms | | 5 |
| **f** | I don't trust my boy/girlfriend? | | 6 |
| **g** | I am sick | | 7 |
| **h** | My partner is sick | | 8 |
| **i** | My boy/girlfriend died of AIDS | | 7 |
| **j** | I had an accident/cuts | | 10 |
| **k** | Other | | 11 |

| **7.3** | **Have you ever had an HIV test?** | Yes | No | Don’t know |
| --- | --- | --- | --- | --- |
|  |  | 1 | 2 | 3 |
|  |  | | ***GO TO 8.1*** | ***GO TO 8.1*** |

| **7.4** | **How long ago did you have your most recent HIV test?** | |
| --- | --- | --- |
| Less than 12 months ago | | 1 |
| 12-23 months ago | | 2 |
| 2 or more years ago | | 3 |
| Don’t know | | 4 |

| **7.5** | **Did you receive the results of your last HIV test?** | | | |
| --- | --- | --- | --- | --- |
| Yes | | No | Don’t know | Refused |
| 1 | | 2 | 3 | 4 |
|  | | ***GO TO 8.1*** | ***GO TO 8.1*** | ***GO TO 8.1*** |

| **7.6** | **Would you be willing to tell me the last HIV test result you received?** | | | |
| --- | --- | --- | --- | --- |
| Yes | | No | Don’t know | Refused |
| 1 | | 2 | 3 | 4 |
|  | | ***GO TO 8.1*** | ***GO TO 8.1*** | ***GO TO 81*** |

| **7.7** | **What were the results of that HIV test?** | | | |
| --- | --- | --- | --- | --- |
| Positive | | Negative | Indeterminate | Don’t know |
| 1 | | 2 | 3 | 4 |
|  | | ***GO TO 8.1*** | ***GO TO 8.1*** | ***GO TO 8.1*** |

| **7.8** | **Have you ever taken medicine, that is, antiretroviral (ARV) medication, to treat HIV infection?** | Yes | No | Don’t know |
| --- | --- | --- | --- | --- |
|  |  | 1 | 2 | 3 |
|  |  | | ***GO TO 8.1*** | |

| **7.9** | **Are you currently taking ARVS, that is antiretroviral medications, daily?** | Yes | No |
| --- | --- | --- | --- |
|  |  | 1 | 2 |

| **SECTION 8** | **ALCOHOL USE.** |
| --- | --- |

| **INSTRUCTION** | ***The next section contains questions on the use of alcohol. Please remember that your name is not written anywhere and everything you tell me is confidential.*** |
| --- | --- |
|  | **USE THE EXAMPLE BELOW TO HELP YOU UNDERSTAND WHAT A STANDARD UNIT OR A STANDARD DRINK IS:** |

| One standard drink:   \| 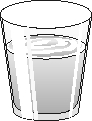 \| *A single tot of spirits(like brandy)*  *(e.g., 25ml at 43%)* \| 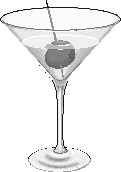 \| *A small glass of liqueur or aperitif*  *(e.g., 25ml at 30%)* \| \| \| \| --- \| --- \| --- \| --- \| --- \| --- \| \| 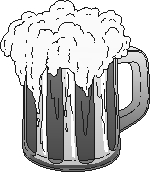 \| *1 can of ordinary beer*  *(e.g., 340ml at 5%)* \| 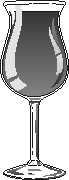 \| *1 glass of wine*  *(e.g., 120ml at 12%)* \| 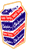 \| *Carton of ordinary commercial sorghum beer (e.g., 500ml at 3%)* \| |
| --- | --- | --- | --- | --- | --- | --- | --- | --- | --- | --- | --- | --- |

| **8.1** | **Have you ever had a drink containing alcohol?** | Yes | No |
| --- | --- | --- | --- |
|  |  | 1 | 2 |
|  |  |  | ***GO TO 8.4*** |

| **8.2** | **How often did you have a drink containing alcohol in the past 12 months?** | | | | |
| --- | --- | --- | --- | --- | --- |
|  | Not in the past 12 months | Once a month or less | 2-4 times a month | 2-3 times a week | 4 or more times a week |
|  | 1 | 2 | 3 | 4 | 5 |
|  | ***GO TO 8.4*** |  | | | |

| **8.3** | **How many drinks containing alcohol do you have on a typical day when you are drinking?** | | | | |
| --- | --- | --- | --- | --- | --- |
|  | 1 or 2 | 3 or 4 | 5 or 6 | 7 to 9 | 14 or more |
|  | 1 | 2 | 3 | 4 | 5 |

| **INSTRUCTION** | | **DO NOT READ OUT OPTIONS. MULTIPLE RESPONSES POSSIBLE** | |
| --- | --- | --- | --- |
| **8.4** | **Where have you seen alcohol adverts?** | | |
| **a** | TV | | 1 |
| **b** | Radio | | 2 |
| **c** | Social media | | 3 |
| **d** | Facebook | | 4 |
| **e** | Twitter | | 5 |
| **f** | Instagram | | 6 |
| **g** | We Chat | | 7 |
| **h** | Mxit | | 8 |
| **i** | Internet | | 9 |
| **j** | Posters | | 10 |
| **k** | Billboards | | 11 |
| **l** | Events | | 12 |
| **m** | Sport grounds, etc. | | 13 |

**Exit questions**

|  | | | |
| --- | --- | --- | --- |
| **8.5** | Thank you, we are almost finished. Was it difficult or easy to do this interview on the computer | **Easy** | **Difficult** |
|  |  | 1 | 2 |

|  | | | | |
| --- | --- | --- | --- | --- |
| **8.6** | Do you prefer such an interview with a person or with a computer? | Prefer a person | Prefer a computer | Both are OK |
|  |  | 1 | 2 | 3 |

|  | | | | |
| --- | --- | --- | --- | --- |
| **8.7** | Are you more likely to tell private things about yourself to a person or a computer? | Prefer a person | Prefer a computer | Both are OK |
|  |  | 1 | 2 | 3 |

|  | |  | | | | | |
| --- | --- | --- | --- | --- | --- | --- | --- |
| **8.8** | What was the main reason for agreeing to participate in this survey? *(Read categories, check all that apply)* | |  | HIV testing | Other testing | Reimbursement | **All mentioned** |
|  |  |  |  | 2 | 3 | 4 | 5 |

If there is another reason not mentioned, please tell me( 6) ……………………………………………

| **INTERVIEW ENDING TIME:** |  |  | : |  |  |
| --- | --- | --- | --- | --- | --- |

***THANK YOU VERY MUCH FOR AGREEING TO PARTICIPATE AND ASSIST US IN THIS IMPORTANT PROJECT.***

### **Appendix 7: Questionnaire for parent/guardian of children aged 0 to 11 years**

**JOINT TUBERCULOSIS AND HIV SURVEY PILOT 2019**

Barcode

Questionnaire number

| **A** | **GEOGRAPHIC AND INTERVIEW PARTICULARS** | | | | | | | | |
| --- | --- | --- | --- | --- | --- | --- | --- | --- | --- |
| Province | | | | | | | | |  |
| Cluster number | | | | |  |  |  |  |  |
| Small area layer | |  |  |  |  |  |  |  |  |
| Visiting point number (from the map) | | | | |  |  |  |  |  |
| Person number of respondent | | | | | | | |  |  |

| **B** | **INTERVIEW DETAILS** | | | | | | | | | |
| --- | --- | --- | --- | --- | --- | --- | --- | --- | --- | --- |
|  | | Year | | Month | | Day | | Time code | Response code |  |
| First visit | | 1 |  |  |  |  |  |  |  |  |
| Second visit | | 1 |  |  |  |  |  |  |  |  |
| Third visit | | 1 |  |  |  |  |  |  |  |  |
| Final response code | | | | | | | | |  |  |
| **Time code**  1 = Morning till 12:00  2 = 12:00-16:00  3 = 16:00-18:00  4 = 18:00-20:00  5 = 20:00 and later | | **Response code**  1 = Interview completed and sample taken  2 = Interview completed but sample not taken  3 = Appointment made for interview and/or sample  4 = Selected respondent not at home  5 = Refusal by head of household  6 = Refusal by respondent  7 = Other | | | | | | | |  |

| **INTERVIEW STARTING TIME:** |  |  | : |  |  |
| --- | --- | --- | --- | --- | --- |

| **INTERVIEWER: NAME AND NUMBER ……………………………………...** |  |  |  |  |
| --- | --- | --- | --- | --- |

| **C** | **REFUSAL PARTICULARS (IF APPLICABLE)** | |
| --- | --- | --- |
| At what point did the respondent refuse?  SPECIFY | |  |

1 = At the reception desk

2 = After explanation of the survey and the process (group information session)

4 = During the individual interview

5 = After the individual interview when requested to do the test

6 = Other

Refusals during individual interview

20 = Objected to providing any/some information on the topic

21 = Objected to providing personal/confidential information

22 = Unable to provide requested information

23 = Refused to continue because he/she got irritated/bored

24 = Refused to continue because he/she got angry

25 = Refused to continue because he/she lost interest or got tired

26 = Refused to continue because he/she was in a hurry

27 = Other

Refusal to provide a blood sample

40 = Apprehensive of blood sample being taken

41 = Against religious beliefs to provide a blood sample

42 = Did not want to know HIV status

43 = Fear a breach of confidentiality

44 = Did not trust the interviewers

45 = Recently had an HIV test

45= Other

| **Collection and use of demographic information after refusal to participate or withdrawal from the study** | | |
| --- | --- | --- |
|  | I agree that you can record and use information on my age, sex, and level of education. | Child  Yes………………………………………..1  No.………………………………………..2  Parent/ guardian  Yes………………………………………..1  No.………………………………………..2 |

| **GENERAL INSTRUCTION** | **CIRCLE THE CODE NEXT TO THE APPROPRIATE ANSWER. IF INDICATED READ THE ANSWER OPTIONS. *Please remember that your name is not written anywhere and everything you tell me is confidential.*** |
| --- | --- |

| **SECTION 1** | **PARENT/GUARDIAN’S DEMOGRAPHIC DATA** |
| --- | --- |

| **1.1** | **Are you the biological parent of this child?** | Yes | No |
| --- | --- | --- | --- |
|  |  | 1 | 2 |
|  | | **GO TO 1.5** |  |

| **1.2** | **Are you the guardian of this child? (i.e. taking care of/take responsibility for this child)** | Yes | No |
| --- | --- | --- | --- |
|  |  | 1 | 2 |
|  | | | **GO TO 1.4** |

| **1.3** | **What is your relationship to this child?** | |
| --- | --- | --- |
| Adoptive parent | | 1 |
| Stepparent | | 2 |
| Grandparent | | 3 |
| Sister/Brother | | 4 |
| Other family (e.g., aunt) | | 5 |
| Unrelated guardian | | 6 |
| Other | | 7 |

| **1.4** | **Where is the parent/guardian of this child?** | |
| --- | --- | --- |
| Unavailable for interview because of work | | 1 |
| Unavailable because of another commitment | | 2 |
| Ill at home or in hospital | | 3 |
| Not living in the same house, but living in the same area | | 4 |
| Living in another area | | 5 |
| Deceased | | 6 |
| Alive, but we don't know where he/she is | | 7 |
| Other (specify): ……………………………………………………………….. | | 8 |

| **INSTRUCTION** | **MAKE AN APPOINTMENT TO INTERVIEW THE PARENT / GUARDIAN. IF THE GUARDIANSHIP IS UNCLEAR, CONSULT WITH YOUR SUPERVISOR** |
| --- | --- |

| **INSTRUCTION** | **HAS THE PARENT / GUARDIAN SIGNED A CONSENT/PERMISSION FORM IN RESPECT OF THIS CHILD?** | Yes | No |
| --- | --- | --- | --- |
| **1.5** |  | 1 | 2 |

| **INSTRUCTION** | **IF NO, GET THE PARENT/GUARDIAN TO SIGN A CONSENT/PERMISSION FORM OR END THE INTERVIEW** |
| --- | --- |

| **INSTRUCTION** | **WHAT IS THE PERSON NUMBER OF THE RESPONDENT COMPLETING THIS QUESTIONNAIRE?** (The person who is responding to the questionnaire. Get the number from the household schedule in the visiting point questionnaire) |  |  |
| --- | --- | --- | --- |
| **1.6** |  |  |  |

| **INSTRUCTION**  **1.7** | **WHAT IS THE PERSON NUMBER OF THE BIOLOGICAL MOTHER OF THIS CHILD?** (Get the person number from the household schedule in the visiting point questionnaire). If the biological mother does not stay in the household or has died, leave blank. |  |  |
| --- | --- | --- | --- |

| **INSTRUCTION**  **1.8** | **WHAT IS THE PERSON NUMBER OF THE BIOLOGICAL FATHER OF THIS CHILD?** (Get the person number from the household schedule in the visiting point questionnaire). If the biological father does not stay in the household or has died, leave blank. |  |  |
| --- | --- | --- | --- |

| **1.9** | **How old were you at your last birthday? (Age of Parent/Guardian in years)** |  |  |
| --- | --- | --- | --- |

| **INSTRUCTION**  **1.10** | **How would you describe yourself?** | Male | Female |
| --- | --- | --- | --- |
|  |  | 1 | 2 |

| **SECTION 2** | **CHILD’S BIOGRAPHICAL DATA. *Please remember that your name is not written anywhere and everything you tell me is confidential.*** |
| --- | --- |

| **2.1a** | **How old is the child? If less than one year enter in age months** | | | | |  | |  | |
| --- | --- | --- | --- | --- | --- | --- | --- | --- | --- |
| **2.1b** | **How old is the child? If one year and older, enter in age years** | | | | |  | |  | |
| **2.1c** | **What is the child’s date of birth** | | | | | | | | |
| Year | | | | Month | |  | | | |
|  | | |  |  |  |  | |  | |

| **2.2** | **What is the sex of the child?** | Male | Female |
| --- | --- | --- | --- |
|  |  | 1 | 2 |

| **2.3** | **Which of the following describes the child’s race?** | | | | |
| --- | --- | --- | --- | --- | --- |
| African | | White | Coloured | Indian/Asian | Other |
| 1 | | 2 | 3 | 4 | 5 |

| **2.4** | **What is the nationality of the child? By that I mean is the child South African or from another country.** | |
| --- | --- | --- |
| South African citizen | | 1 |
| Non-citizen (Permanent resident ) | | 2 |
| Non-citizen (Refugee) | | 3 |
| Other | | 4 |

| **INSTRUCTION** | **ONLY ASK IF YOU ARE NOT INTERVIEWING THE BIOLOGICAL MOTHER** |
| --- | --- |

| **2.5** | **Is the child’s biological mother alive?** | Yes | No | Don’t know |
| --- | --- | --- | --- | --- |
|  |  | 1 | 2 | 3 |
|  | | ***GO TO 2.7*** |  | ***GO TO 2.7*** |

| **2.6** | How old was the child when she passed away? | | |
| --- | --- | --- | --- |
| **2.6a** | **If less than one year enter AGE in months** |  |  |
| **2.6b** | **If one year and older, enter AGE in years** |  |  |

| **2.6c** | **IF RESPONDENT DOES NOT KNOW THE AGE WHEN SHE DIED, PLEASE CIRCLE ‘3’’** | 3 |
| --- | --- | --- |

| **INSTRUCTION** | **ONLY ASK IF YOU ARE NOT INTERVIEWING THE BIOLOGICAL FATHER** |
| --- | --- |

| **2.7** | **Is the child’s biological father alive?** | Yes | No | Don’t know |
| --- | --- | --- | --- | --- |
|  |  | 1 | 2 | 3 |
|  | | ***GO TO 2.9*** |  | ***GO TO 2.9*** |

| **2.8** | **How old was the child when he passed away?** | | |
| --- | --- | --- | --- |
| **2.8a** | **If less than one year enter AGE in months** |  |  |
| **2.8b** | **If one year and older, enter AGE in years** |  |  |

| **2.8c** | **IF RESPONDENT DOES NOT KNOW THE AGE WHEN HE DIED, PLEASE CIRCLE ‘3’** | 3 |
| --- | --- | --- |

| **2.9** | **Does the child have a birth certificate?** | Yes | No | Don’t know |
| --- | --- | --- | --- | --- |
|  |  | 1 | 2 | 3 |

| **SECTION 3** | **HEALTH STATUS AND HEALTH QUESTIONS** |
| --- | --- |

| **INSTRUCTION** | ***Now I am going to ask you questions about this child’s health as well as about the services this child receives in clinics, hospitals or elsewhere. Please remember that your name is not written anywhere and everything you tell me is confidential.*** |
| --- | --- |

| **3.1** | **In general, would you say that this child’s health is excellent, good, fair or poor?** | |
| --- | --- | --- |
| Excellent | | 1 |
| Good | | 2 |
| Fair | | 3 |
| Poor | | 4 |

| **3.2** | **When was the last time this child went to see a healthcare worker (such as doctor, nurse, clinic sister)?** | |
| --- | --- | --- |
| Within the past six months | | 1 |
| More than six months but not more than a year ago | | 2 |
| More than a year ago | | 3 |
| Never | | 4 |

| **3.3** | **Where does this child usually obtain health care?** | |
| --- | --- | --- |
| Government hospital | | 1 |
| Day hospital/clinic community health centre/well baby clinic | | 2 |
| Mobile clinic | | 3 |
| Private hospital/clinic | | 4 |
| Other private medical | | 5 |
| Traditional healer | | 6 |
| School clinic | | 7 |
| Other | | 8 |

| **3.4** | **In the last 12 months, has this child been hospitalized for any illness? Admitted at least one night** | Yes | No |
| --- | --- | --- | --- |
|  |  | 1 | 2 |
|  |  |  | ***GO TO 3.7*** |

| **3.5** | **How many times has this child been admitted to hospital during the past 12 months?** |  |  |
| --- | --- | --- | --- |

| **3.6** | **What was the total time this child has spent in hospital during the last 12 months? (In days)** |  |  |  |
| --- | --- | --- | --- | --- |

| **INSTRUCTION** | **ONLY ASK “Number of times” IF CHILD WAS TAKING TO A HEALTH CARE PROVIDER** |
| --- | --- |

| **3.7** | **In the last 12 months, to which of the following health care providers has this child been taken and how many times in all?** | **Yes** | **No** | **Number of times** |
| --- | --- | --- | --- | --- |
| **a** | Government hospital | 1 | 2 |  |
| **b** | Day hospital/clinic /community health centre | 1 | 2 |  |
| **c** | Mobile clinic | 1 | 2 |  |
| **d** | Private hospital/clinic | 1 | 2 |  |
| **e** | Pharmacy | 1 | 2 |  |
| **f** | Private doctor | 1 | 2 |  |
| **g** | Other private medical | 1 | 2 |  |
| **h** | School clinic | 1 | 2 |  |
| **i** | Traditional Healers | 1 | 2 |  |
| **j** | Other | 1 | 2 |  |

| **INSTRUCTION** | ***The next section deals with circumcision*** | |
| --- | --- | --- |
| **MALE CHILD** | | **Q4.7**  **FEMALE CHILD** |

| **SECTION 4** | **MALE CIRCUMCISION. *Please remember that your name is not written anywhere and everything you tell me is confidential.*** |
| --- | --- |

| **4.1** | **Has the child been circumcised?** | Yes | No | Don’t know |
| --- | --- | --- | --- | --- |
|  |  | 1 | 2 | 3 |
|  | | | **GO TO 4.6a** | **GO TO 4.6a** |

| **4.1a** | **Type of circumcision?** | |
| --- | --- | --- |
| Partial (Some foreskin remains) | | 1 |
| Full/complete (Foreskin totally removed) | | 2 |
| Don’t know | | 3 |

| **4.2** | **How old was the child when he was circumcised?** | | |
| --- | --- | --- | --- |
| **4.2a** | **If child is less than one year, enter AGE in months** |  |  |
| **4.2b** | **If child one year and older, enter AGE in years** |  |  |

| **4.3** | **What was your main reason for circumcising this child?** | |
| --- | --- | --- |
| Traditional practice such as initiation | | 1 |
| Religious reasons | | 2 |
| Health reasons | | 3 |
| Prevent HIV and other STIs | | 4 |
| Other | | 5 |

| **4.3a** | **Where was the child circumcised?** | |
| --- | --- | --- |
| At home | | 1 |
| In hospital/clinic | | 2 |
| In the mountains/in the bush/initiation school | | 3 |
| Circumcision camp | | 4 |
| Mobile clinic/circumcision clinic | | 5 |
| Other | | 6 |
| Don’t know | | 7 |

| **4.4** | **Who performed the circumcision?** | |
| --- | --- | --- |
| Doctor | | 1 |
| Spiritual or religious leader | | 2 |
| Traditional circumciser | | 3 |
| Other | | 4 |
| Don’t know | | 5 |

| **4.5** | **Did the child experience any complications following circumcision?** | Yes | No | Don’t know |
| --- | --- | --- | --- | --- |
|  |  | 1 | 2 | 3 |
|  | | ***GO TO 4.7*** | ***GO TO 4.7*** | ***GO TO 4.7*** |

| **INSTRUCTION** | **ASK IF CHILD IS NOT CIRCUMCISED** | Yes | No | Unsure |
| --- | --- | --- | --- | --- |
| **4.6a** | Would you consider circumcision for your male child? | 1 | 2 | 3 |
|  | | ***GO TO 4.7*** |  | ***GO TO 4.7*** |

| **4.6b** | **Why would you not consider male circumcision?** | |
| --- | --- | --- |
| Traditional reasons | | 1 |
| Religious reasons | | 2 |
| Health reasons | | 3 |
| Other | | 4 |

| **4.7** | **Was the child ever scarified in any part of the body? (*Scarification is a traditional practice where children are scarred using the razor blade and blood is usually mixed with African medicine to prevent them from being bewitched or getting sick or to make them stronger)*** | Yes | No | Don’t  know |
| --- | --- | --- | --- | --- |
|  |  | 1 | 2 | 3 |

| INSTRUCTION | **AGE FILTER** | |
| --- | --- | --- |
| **CHILDREN AGED 5 YEARS AND**  **OLDER:** | | **Q 6.1**  **CHILDREN YOUNGER**  **THAN 5 YEARS:** |

| **SECTION 5** | **SCHOOL ATTENDANCE OF THE CHILD** |
| --- | --- |

| **INSTRUCTION** | ***The next questions I am going to ask you relate to the school attendance of the child. Please remember that your name is not written anywhere and everything you tell me is confidential.*** |
| --- | --- |

| **5.1a** | **Does the child attend school?** | Yes | No |
| --- | --- | --- | --- |
|  |  | 1 | 2 |
|  | | | ***GO TO 5.1c*** |

| **5.1b** | **What Grade is the child attending this year?** | |
| --- | --- | --- |
| Pre-school/ Gr R | | 0 |
| Grade 1/Sub a/Class 1 | | 1 |
| Grade 2/Sub b/Class 2 | | 2 |
| Grade 3/Standard 1/Abet 1 | | 3 |
| Grade 4 /Standard 2/Abet 2 | | 4 |
| Grade 5 /Standard 3/Abet 2 | | 5 |
| Grade 6 /Standard 4/Abet 3 | | 6 |
|  | | ***GO TO 6.1*** |

| **5.1c** | **If ‘No’, what is the highest grade the child completed successfully at school?** | |
| --- | --- | --- |
| Pre-school/ Gr R | | 0 |
| Grade 1/Sub a/Class 1 | | 1 |
| Grade 2/Sub b/Class 2 | | 2 |
| Grade 3/Standard 1/Abet 1 | | 3 |
| Grade 4 /Standard 2/Abet 2 | | 4 |
| Grade 5 /Standard 3/Abet 2 | | 5 |
| Never attended school | | 6 |

| **SECTION 6** | **EDUCATION OF THE CHILD ON LIFE ISSUES *Please remember that your name is not written anywhere and everything you tell me is confidential.*** |
| --- | --- |

| **INSTRUCTION** | ***The next questions I am going to ask you relate to the information and education the child receives at home***. |
| --- | --- |

| **6.1** | **Have you ever discussed sex with this child**? | Yes | No | No response |
| --- | --- | --- | --- | --- |
|  |  | 1 | 2 | 3 |

| **6.2** | **Have you ever discussed sexual abuse with this child**? | Yes | No | No response |
| --- | --- | --- | --- | --- |
|  |  | 1 | 2 | 3 |

| **SECTION 7** | **HIV TESTING AND MEDICATION** |
| --- | --- |

| **INSTRUCTION** | ***I have some more questions about this child. Some of these are about HIV. Your answers will not be told to anyone outside the study. They will not be told to this child or anyone else in your family.*** |
| --- | --- |

| **7.1** | **Has the child ever been tested for HIV?** | | Yes | No | No response |
| --- | --- | --- | --- | --- | --- |
|  |  |  | 1 | 2 | 3 |
|  |  | | | ***GO TO NEXT AGE FILTER*** | |

| **7.2** | **How long ago was the child’s last HIV test?** | |
| --- | --- | --- |
| 0 to 3 months | | 1 |
| 4 to 6 months | | 2 |
| 7 to 11 months | | 3 |
| Less than a year ago | | 4 |
| Between 1-2 years ago | | 5 |
| Between 2-3 years ago | | 6 |
| Three or more years ago | | 7 |

| **7.3** | **Are you willing to disclose the child’s HIV test results?** | Yes | No |
| --- | --- | --- | --- |
|  |  | 1 | 2 |
|  |  |  | ***GO TO 8.1*** |

| **7.4** | **What was the child’s last HIV test result?** | Positive | Negative | Indeterminate | I did not receive result |
| --- | --- | --- | --- | --- | --- |
|  |  | 1 | 2 | 3 | 4 |
|  | | | ***GO TO 8.1*** | | |

| **7.5** | **Has the child** been told that he/she is infected with **HIV?** | | Yes | No | Don’t know |
| --- | --- | --- | --- | --- | --- |
|  |  |  | 1 | 2 | 3 |

| **7.6** | **Has this child ever taken medicine, that is, antiretroviral medication, to treat HIV infection?** | Yes | No | Don’t know |
| --- | --- | --- | --- | --- |
|  |  | 1 | 2 | 3 |
|  |  |  | ***GO TO NEXT AGE FILTER*** |  |

| **7.7** | **Is the child currently taking ARVs, that is, antiretroviral medications, daily?** | Yes | No |
| --- | --- | --- | --- |
|  |  | 1 | 2 |
|  | | ***GO TO NEXT AGE FILTER*** |  |

| **7.8** | **Can you tell me the main reason why the child** is **not taking ARVs daily?** | |
| --- | --- | --- |
| Have trouble giving the child ARVs everyday | | 1 |
| The child had side effects/ARVs made the child sick | | 2 |
| Facility/pharmacy too far away to get ARVs regularly | | 3 |
| I can’t afford/ ARVs, too expensive | | 4 |
| I don’t think the child needs ARVs, the child is not sick | | 5 |
| Pharmacy/facility was out of stock of ARVs | | 6 |
| I fear people will know that the child has HIV, if ARVs are given | | 7 |
| Too busy/no time to give | | 8 |
| Doctor has not recommended ARVs | | 9 |
| Don’t know | | 10 |
| Other | | 11 |

| INSTRUCTION | **AGE FILTER** | |
| --- | --- | --- |
| **CHILDREN 0 - 2 YEARS** | | **END INTERVIEW**  **CHILDREN 3 – 11 YEARS** |

| INSTRUCTION | | **BIOLOGICAL MOTHER OF THIS CHILD FILTER** | |  |
| --- | --- | --- | --- | --- |
| **BIOLOGICAL MOTHER** | | | **END INTERVIEW**  **CARE GIVER, ADOPTIVE**  **PARENT, ETC.** |  |
| **SECTION 8** | **ANTE- AND POST-NATAL CARE AND INFANT FEEDING.** | | | |

| **INSTRUCTION** | ***Now I would like to ask you about the pregnancy and feeding of this child Please remember that your name is not written anywhere and everything you tell me is confidential..*** |
| --- | --- |

| **8.1** | **Where was the child born?** | Hospital | Clinic | Home or other | Don’t Know |
| --- | --- | --- | --- | --- | --- |
|  |  | 1 | 2 | 3 | 4 |

| **8.2** | **Who attended the birth of the child?** | Doctor | Nurse / Midwife (health care worker) | Traditional birth attendant or other | Don’t Know |
| --- | --- | --- | --- | --- | --- |
|  |  | 1 | 2 | 3 | 4 |

| **8.3** | **Did you see anyone for antenatal care for this pregnancy?** | **Yes** | **No** |
| --- | --- | --- | --- |
|  |  | 1 | 2 |
|  | | | ***GO TO 8.8*** |

| **8.4** | **Whom did you see?** | |
| --- | --- | --- |
| Doctor | | 1 |
| Nurse/Midwife | | 2 |
| Traditional birth attendant | | 3 |
| Other | | 4 |

| **8.5** | **Where did the first antenatal visit take place?** | |
| --- | --- | --- |
| Government hospital | | 1 |
| Day hospital/clinic/community health centre | | 2 |
| Mobile clinic | | 3 |
| Private hospital/clinic | | 4 |
| Other private medical facility | | 5 |
| Other | | 6 |

| **8.6a** | **How many weeks pregnant were you when you first received antenatal care for this pregnancy?** | Weeks | Don’t know |
| --- | --- | --- | --- |
|  |  |  |  |

| **8.6b** | **How many visits did you make to the antenatal care clinic during this pregnancy?** |  |  |
| --- | --- | --- | --- |

| **8.7** | **During this pregnancy, were any of the following done at least once?** | Yes | No | Don’t know |
| --- | --- | --- | --- | --- |
| 1. **e** | Did you give a blood sample? | 1 | 2 | 3 |
| 1. **g** | Have you received information on HIV transmission? | 1 | 2 | 3 |
| 1. **h** | Were you tested for HIV? | 1 | 2 | 3 |
| 1. **j** | Were you tested for sexually transmitted diseases, including syphilis? | 1 | 2 | 3 |
| 1. **k** | Were you given advice on nutrition? | 1 | 2 | 3 |
| 1. **l** | Were you given an ultrasound? | 1 | 2 | 3 |

| **INSTRUCTION** | | ***I am now going to ask you a number of questions concerning the feeding practices of the child*** | | |
| --- | --- | --- | --- | --- |
| **8.8** | **Did you ever breastfeed the baby?** | | **Yes** | **No** |
|  |  |  | 1 | 2 |
|  | | | | ***GO TO 8.12*** |

| **8.9** | **How long after birth did you first put the baby on the breast?** | **Hours** | | **Days** | |
| --- | --- | --- | --- | --- | --- |
|  |  |  |  |  |  |

| **8.10** | **Did you ever exclusively breastfeed this baby? Exclusive breastfeeding is giving your baby breast milk only.** | **Yes** | **No** |
| --- | --- | --- | --- |
|  |  | 1 | 2 |
|  |  |  | ***GO to 8.12*** |

| **8.11** | **If yes, for how long did you exclusively breastfeed this baby?** | |
| --- | --- | --- |
| Less than one month | | 1 |
| Two months up to six months | | 2 |
| More than six months | | 3 |

| **8.12** | **Did you ever give your baby other foods during the first six months after birth?** | **Yes** | **No** |
| --- | --- | --- | --- |
|  |  | 1 | 2 |

| **8.13** | **Did you feed this baby formula during the first six months after birth?** | **Yes** | **No** |
| --- | --- | --- | --- |
|  |  | 1 | 2 |

| **8.14** | **Was there a time when no formula was available?** | **Yes** | **No** | **Don’t know** |
| --- | --- | --- | --- | --- |
|  |  | 1 | 2 | 3 |
|  |  | | ***GO to 8.16*** | ***GO to 8.16*** |

| **INSTRUCTION** | **DO NOT READ OUT OPTIONS. MULTIPLE RESPONSES POSSIBLE** |
| --- | --- |

| **8.15** | **How was the baby fed during this time?** | |
| --- | --- | --- |
| **a** | Breast milk | 1 |
| **b** | Fresh cow’s milk, tea with milk, weak porridge/ goat’s milk | 2 |
| **c** | Water only, water with sugar/glucose, fruit juice, tea without milk, rice water | 3 |
| **d** | Solid food (e.g., yoghurt, cheese, cereals, porridge, bread, fermented porridge, fruits/vegetables, meat/fish/chicken, eggs) | 4 |
| **e** | Other | 5 |

| **8.16** | **If formula fed, where was the formula usually obtained?** | |
| --- | --- | --- |
| Clinic | | 1 |
| Store | | 2 |
| Other | | 3 |
| Never formula fed | | 4 |

| **8.17** | **Was this baby ever fed breast milk from another woman?** | **Yes** | **No** |
| --- | --- | --- | --- |
|  |  | 1 | 2 |

**9Exit questions**

|  | | | |  |
| --- | --- | --- | --- | --- |
| **9.1** | Thank you, we are almost finished. Was it difficult or easy to do this interview on the computer | ***Easy*** | ***Difficult*** | **N/A** |
|  |  | 1 | 2 | 4 |

|  | | | | | |  |
| --- | --- | --- | --- | --- | --- | --- |
| **9.2** | Do you prefer such an interview with a person or with a computer? | Prefer a person | Prefer a computer | | Both are OK | N/A |
|  |  | 1 | 2 | 3 | | 4 |

|  | | | | |  |
| --- | --- | --- | --- | --- | --- |
| **9.3** | Are you more likely to tell private things about your child to a person or a computer? | Prefer a person | Prefer a computer | Both are OK | N/A |
|  |  | 1 | 2 | 3 | 4 |

|  |  | | | | | |
| --- | --- | --- | --- | --- | --- | --- |
| **9.4** | What was the main reason for agreeing to take part in this survey? *(Read categories, check all that apply)* |  | HIV testing | Other testing | Reimbursement | **All mentioned** |
|  |  |  | 2 | 3 | 4 | 5 |

If there is another reason not mentioned, please tell me (6)……………………………………………

***THANK YOU VERY MUCH FOR AGREEING TO TAKE PART AND ASSISTING US IN THIS IMPORTANT RESEARCH PROJECT.***

| **INTERVIEW ENDING TIME:** |  |  | : |  |  |
| --- | --- | --- | --- | --- | --- |

### **Appendix 8: Qualitative Measures: Key Informant and Focus Group Guide**

***Joint TB and HIV Impact assessment pilot survey***

| **1. Tell me about the nature of your involvement in the study?**  Probes: What was your role? Did the roles fit with your skills? |
| --- |
| **2. Please share with me some of the highlights from the project?**  **Probes**: What did the study do well?  (Explore this question for the different aspects of the study training, entry, recruitment of participants, processes at the hub, implementation and data collection, testing HIV and TB, transportation of samples, communication and study mobilization, lab aspects) |
| **3. Please share with us some of the challenges you experienced during the implementation of the project, low points or areas that the project could improve?**  **Probe**: What did not work well? Which aspect of the study were the most challenging?  (Explore this question for the different aspects of the study training, entry, recruitment of participants, processes at the hub, implementation and data collection, testing HIV and TB; transportation of samples, communication and study mobilization, lab aspects) |
| **4. Looking back at the project, tell me what were the gaps that you identified?**  **Probes:** What gaps did you come across?  (Explore this question for the different aspects of the study training, entry, recruitment of participants, processes at the hub, implementation and data collection, testing HIV and TB; transportation of samples, communication and study mobilization, lab aspects) |
| **5. Looking at the challenges and gaps you have mentioned to me, can you now tell me what needs to be improved or done better if we were to implement this project again?**  **Probes:** What lessons did you learn? – (focus more on the solutions and future surveys for this questions - if already covered under question 3 this question can be let out)  (Explore this question for the different aspects of the study training, entry, recruitment of participants, processes at the hub, implementation and data collection, testing HIV and TB; transportation of samples, communication and study mobilization, lab aspects) |

## Appendix 9: TB Suspect Register

***Joint Tuberculosis and HIV Pilot Survey***

**Province:** ____________________________

**District: ____________**____________

**Cluster Name: ____________**____________

**Cluster Code: ____________**_____________

| Date of Cluster work |  |  | to | |  |  |
| --- | --- | --- | --- | --- | --- | --- |
| **Date completed**  **dd/mm/yy** |  |  |  |  |  |  |

| **Household Number** | **Barcode (unique identifier)** | **Name (Use Capitals)** | **Suspect on symptoms** | **Suspect on X-ray** | **1^st^ specimen** | **Results of 1^st^ specimen** | **2^nd^ specimen** | **Results of 2^nd^ specimen** | **TB Case** | **Identification** |
| --- | --- | --- | --- | --- | --- | --- | --- | --- | --- | --- |
| ### | ### |  | y/n | y/n | y/n | N/S/P | y/n | N/S/P | Tick | TB/MOTT |
|  |  |  |  |  |  |  |  |  |  |  |
|  |  |  |  |  |  |  |  |  |  |  |
|  |  |  |  |  |  |  |  |  |  |  |

## Appendix 10: Field Measurement and Biomarker Form

***Joint Tuberculosis and HIV Pilot Survey***

Barcode:

| ***Weight** |  |  | **Kg** |
| --- | --- | --- | --- |

| ***Height** |  |  | **metres** |
| --- | --- | --- | --- |

| ***Random blood glucose** |  |  | **mmol/L** |
| --- | --- | --- | --- |

| ***Cholesterol** |  |  | **mg/dL** |
| --- | --- | --- | --- |

***Only if 18years and older**

**HIV testing**

| **Pre- and post-counselling for HIV completed?** | **Yes** |  | **No** |  |
| --- | --- | --- | --- | --- |

| **Rapid HIV test result?** | **Negative** |  | **Positive** |  | **Inconclusive** |  |
| --- | --- | --- | --- | --- | --- | --- |

## Appendix 11: Laboratory Results Retrieval Form

***Joint Tuberculosis and HIV Impact Assessment Pilot Survey*:**

Barcode:

**This individual participated in the joint TB and HIV Impact Assessment Pilot Survey. Results of some of the tests have been sent to the clinic.**

| **Province** | **EC** | **FS** | **GP** | | **KZN** | **LP** | **MP** | **NC** | **NW** | **WC** |
| --- | --- | --- | --- | --- | --- | --- | --- | --- | --- | --- |
| Age |  | | |  | | | | | | |
| Sex |  | | |  | | | | | | |

| D | D | M | M | Y | Y | Y | Y |
| --- | --- | --- | --- | --- | --- | --- | --- |

**Date from when results can be**

**Retrieved**
