# Supplementary material for: Piloting the feasibility of a population-based joint TB-HIV survey in KwaZulu-Natal Province, South Africa, 2019
Source: PLOS Glob Public Health. 2026 Jan 28;6(1):e0005804. doi: 10.1371/journal.pgph.0005804 (PMC12851483; doi:10.1371/journal.pgph.0005804)
Supplement: S3 Text — (DOCX) [file pgph.0005804.s003.docx]

S3 Data Dictionary: Pilot joint TB-HIV survey in South Africa, 2019

| **Variable / Field Name** | **Field Label**  **Field Note** | **Field Attributes (Field Type, Validation, Choices, Calculations, etc.)** |
| --- | --- | --- |
| **Household data dictionary** | | |
| startdate | Listing date | text (date_dmy) |
| Province | Province | text |
| ClusterNumber | Cluster number | 1. Urban 2. Rural |
| totalnumberofpeoplet hatareinvite | Total number of people that are invited | text (number) |
| age | Age of listed household member | text (number) |
| eligibilityn | Eligibility for survey participation | 1. No 2. Yes |
| invite | Response to survey invitation | Accepted  Declined |
| sexn | Sex of listed household member | Female  Male |
| **Individual data dictionary** | | |
| jths_cluster_name | Cluster name | 1. Urban 2. Rural |
| jths_age_identifier | Age Identifier | text (number) |
| sex | Sex | 1. Male 2. Female |
| jths_15_q3_14 | Any cough | 1. Yes 2. No |
| jths_15_q3_16 | Fever | 1. Yes 2. No |
| jths_15_q3_18 | Drenching night sweats | 1. Yes 2. No |
| jths_15_q3_19 | Unintentional weight loss | 1. Yes 2. No |
| jths_weight | Weight | text (number, Min: 1, Max: 999) |
| jths_height | Height | text (number, Min: 1, Max: 999) |
| jths_bmi | BMI | calc  Calculation:  [jths_weight]*10000/([jths_height]*[jths_height]) |
| jths_bp | Blood Pressure | text |
| jths_glucose | Random Blood Glucose | text (number, Min: 1, Max: 999) |
| jths_cholestrol | Cholesterol | text (number, Min: 1, Max: 999) |
| jths_sample_date_collect | Date of sample collection for HIV testing | text (date_dmy) |
| jths_hiv_venous | Venous blood sample | 1. Yes 2. No |
| jths_hiv_dbs_prick | Finger-prick for DBS specimen | 1. Yes 2. No |
| jths_hiv_rapid | Finger-prick for rapid testing (RBG and cholesterol) | 1. Yes 2. No |
| jths_hiv_mmol | Random blood glucose | text |
| jths_hiv_cholestrol | Cholesterol | text |
| jths_hiv_rapidhiv | Do you want to do rapid HIV test? | 1. Yes 2. No |
| jths_hiv_result |  | 1. Negative 2. Positive 3. Inconclusive |
| jths_cxr_taken | Has CXR been taken | 1. Yes 2. No |
| jths_cxr_date | Date of X-ray: | text (date_dmy) |
| jths_chx_findings | X-RAY Findings | 1. Normal 2. Abnormal -suggestive of TB 3. Abnormal other 4. CXR not taken |
| sput_elig | Sputum eligibility | 1. Symptoms only 2. CXR only 3. Symptoms & CXR 4. CXR exempt |
| jths_sputum_date | Todays Date | text (date_dmy), Required |
| jths_gxp_q1 | Was the spot sputum specimen collected (GXP) | 1. Yes 2. No |
| jths_culture_q3 | Was the morning sputum specimen collected? (CULTURE) | 1. Yes 2. No |
| jths_sam1_qua1 | Sample1 Specimen rejected | 1. Yes 2. No |
| jths_gx_result | Sample (Gene Xpert) Result | 1. MTB detected 2. MTB not detected 3. Invalid 4. trace |
| jths_rifampicin | Rifampicin Resistance | 1. Rifampicin resistance detected 2. Rifampicin resistance not detected 3. Rifampicin indeterminate |
| jths_ge_date | Gene Xpert Date of result | text (date_dmy) |
| jths_hiv_res_final_result | HIV FINAL RESULT | 1. Negative 2. Positive 3. Weak positive 4. Intermediate |
| Viralloadgroupgt1000or_1000copie | Viral load \| group \|  &gt;1000 or \| 1000 \|  copies/ml | 1. <1000 2. 1000 3. ≥1000 |
| FinalSerologyResultGlobalResult | Final Serology Result (Global Result) | Positive  “.” |
| jths_central_image_qua | Image quality | 1. optimal 2. suboptimal 3. unreadable |
