# Supplementary material for: Piloting the feasibility of a population-based joint TB-HIV survey in KwaZulu-Natal Province, South Africa, 2019
Source: PLOS Glob Public Health. 2026 Jan 28;6(1):e0005804. doi: 10.1371/journal.pgph.0005804 (PMC12851483; doi:10.1371/journal.pgph.0005804)
Supplement: S4 Text — (DOCX) [file pgph.0005804.s004.docx]

**S4 Supplementary information**

*Sample size calculation for a full-scale joint TB-HIV survey*

Combining investigations of TB and HIV into a single cross-sectional population-based household survey provides possibilities for better understanding these interrelated epidemics but with several methodological challenges. We attempted to design a hypothetical national sampling frame and design to meet both TB and HIV survey objectives, while also obtaining an overlapping sample of individuals for the analysis of comorbidities, using input parameters suitable for South Africa.

*Geographies and Sample Design Objectives*

Given the difference in prevalence, 0.33% for active TB and 20.6% for HIV among adults (and, possibly, more importantly, clustering effects) different geographies are needed. The typical geography used in most health surveys is the enumeration area (EA), often defined as an area covering 80–120 households, with a target of 100 households (typically redefined at the time of the census). EAs are too small for TB surveys where a minimum of 400 adults are needed, per cluster. An appropriate geographic unit for TB prevalence surveys for South Africa is the small area layer (SAL), which combines EAs with a population of less than 500 with adjacent EAs. In the case of a TB survey, a minimum of 184 households (with an average of 2.72 adults aged 15+ years), would be needed for a cluster ‘take’ of 500 adults. A two-stage approach would be appropriate, beginning with the selection of SALs followed by a selection of households and taking all eligible individuals. For an HIV survey, a three-stage approach might be taken, starting with SALs, followed by EAs, and then households, and taking all eligible individuals in the household. Alternatively, the 2nd stage sample for the HIV component could select from all the households selected for a TB survey as the ‘take’ per cluster for HIV are much smaller (related to design effects). Currently, an average of 35 households are selected from each EA for HIV surveys. Taking a larger number of households per cluster increases the design effects, depending on the outcome of interest. A sample for the HIV component would require a subset of households (70 proposed here) per cluster, and a larger number of clusters.

*Hypothetical sample allocations, estimated design effects, and sample size for a national joint TB-HIV survey*:

i) HIV: Viral load suppression (VLS) at provincial level with 95% CI of +/- 10%; ii) HIV: National incidence with a relative standard error (RSE) of 30% or less; iii) TB: National prevalence with RSE of 12.5% or less (a ‘relative precision’ ‘d’ of 0.25)

HIV surveys have both national (HIV incidence) and sub-national (VLS) objectives. The first round of PHIAs(17,31) (2015–2019) national HIV incidence estimates and subnational VLS dictated sample size requirements. In the second round of the PHIAs, with inputs derived from PHIA1 surveys and new assumptions, the role of VLS at the subnational level gained overall importance in sampling design and allocation, also because HIV incidence estimates continued to decline and hence drive up the respective sample size requirements. When national-level indicators are of central interest (as with TB), proportional (to population) designs are typically best, in that they keep design effects (DEFFs) lower; where subnational estimates are of interest, an equal-size (equal allocation between subnational units (SNUs)) are typically best but result in, at times, much larger design effects for national-level indicators. The impact on design effects for national-level indicators is dependent on the heterogeneity of the HIV epidemic that informs sample allocation by provinces. The allocation of the sample in HIV surveys tend toward non-proportional, more equal-size design, reflecting the heterogeneity of the epidemic (e.g., South Africa).

Given the competing demands associated with TB and HIV surveys a combined sample strategy, with different geographies and ‘takes’, would need to be employed where only a portion of overlap would be possible (likely between 25%-40%, and dependent on the average size of SALs and number of survey hubs available and TB-HIV clusters that could be visited).

**Inputs:** Inputs for the sample design are primarily from the SABSSM 2017 survey (response rates, adults per household, provincial HIV prevalence, national HIV incidence)(11) and, for TB design inputs, from the National TB Prevalence Survey report sampling design discussion and TB prevalence estimates based on 2013 case notification surveillance (in same report)(18). The sample size calculations utilise the following assumptions: i) **Prevalence** – TB prevalence: 333/100 000 or 0.333%; HIV prevalence: 20.5% (ranging from 12.6% in Western Cape to 27.0% in KwaZulu-Natal); HIV incidence: 0.79%; VLS among HIV-infected: 0.5 or 50% (a ‘conservative’ estimate approach for targets); ii) **Clustering Effects – i**ntraclass correlation coefficient (ICC) of 0.05 for VLS; intraclass correlation coefficient (ICC) of 0.000835 for TB prevalence; intraclass correlation coefficient (ICC) of 0.0 for HIV incidence; iii) **Specific to HIV recency testing –** proportion false recent (PFR) = 0.000001%; an adjustment factor 1.016 to account for MDRI and PFR is included for national HIV incidence estimation and associated variance calculations.

The actual number of selected dwelling units (DUs) per cluster will reflect changes in the measure of size between the sampling frame and HH listing. The average number of de facto household members of 3.88 and adults 15 years and older of 2.72, which is based on the SABSSM 2012 survey(10) (similar values found in SABSSM 2017).

A survey uptake of 63.6% among adult participants was assumed (based on interview response of 92.2% and agreeing to a blood draw of 69.0% among those interviewed, based on SABSSM 2017 survey)(11). Detailed sample size calculation is described elsewhere(32).

**Sample size for a full-scale joint TB-HIV survey**

A sample of 122 primary sampling units (PSUs) will be sufficient to meet TB-HIV survey objectives of a) provincial VLS estimates with a 95%CI of +/-10%, b) national HIV incidence estimate with an RSE of 30% or less and c) a national TB prevalence with a 0.25 relative precision. Sixty-four (64) PSUs (SALs) with 280 DUs (to be sampled following a household (HH) listing), is expected to yield near 14 000 HHs responding, with around 24 000 adults (15+) agreeing to TB-HIV testing. Based on an initial estimate of TB prevalence of 0.00333 we can anticipate a 95%CI of +/-0.00085 (0.25% to 0.42%), or a relative precision of 0.25, or an RSE of 12.8%.

In the proposed design are a total of 122 SALs and 64 SALs for the TB-HIV component and an additional 58 SAL-EAs (using a 3-stage sampling strategy: SAL-> EA -> HHs) exclusively for the HIV component. In the 64 SALs selected for TB-HIV components, 280 DUs/HHs would be selected with approximately 760 eligible adults. Following a complete household listing (needed for evaluation of changes in the probability of selections and calculation of weights), DUs/HHs for TB-component would be selected followed by a selection of 70 DUs/HHs for the HIV component (from among the 280). All eligible adults from the 280 DUs/HHs would be eligible for TB component, but only eligible adults from the 70 DUs/HHs would be eligible for both TB and HIV components. As noted above, 58 SAL-EAs of the 122 SAL would be drawn (disproportionally by strata to meet sampling objectives) for only HIV component of the survey.

A subsample of 70 (of 280 DUs) would be required from these households in these 64 SALs and combined with 70 DUs from each of 58 SAL-EAs sampled for the HIV-component of the survey. The HIV-component of the survey would be carried out in a total of 122 PSUs, with an expected 6 631 responding household and 11 451 persons (aged 15+ years) agreeing to a blood draw and testing. This sample is expected to capture 1 735 HIV infected individuals aged 15–49 years, with at least 150 or more in each province, and should provide an estimate of VLS with a 95% CI of +/-10% or less. At national level, the estimated 95% CI for VLS would be around 0.036 (or 3.6%). Based on these sample calculations, we would expect a national HIV incidence estimate with an RSE of 27% (95% CI of 0.38% to 1.2%).

**Households per cluster and response rates:** The average number of selected DUs per cluster is 280 for TB PSUs and 70 for HIV only PSUs, which should yield an average of approximately 217 and 54 responding households per cluster, respectively (based on 77.7% overall household response [94.8% occupancy rate X 82.2% household response]).

An independent TB survey requires 64 SALs with an expected 13 915 responding households, 24 028 responding participants (aged 15+ years). All 64 SALs would be included in the 2^nd^ stage sample of EAs in the HIV component of the combined survey. There is no impact on the TB sample by combining with a HIV survey. An independent HIV survey requires an additional 58 EAs for a total of 120 EAs (from equivalent number of SALs) with an expected 6 285 responding households, and 8 295 adults aged 15–49 years agreeing to participate in the study, with an expected 1 641 HIV positive individuals (aged 15–59 years). Two additional EAs are required in the combined survey given differences in allocations of sample under the different sampling strategies (proportional for TB survey versus provincial VLS approach for HIV surveys). Combining surveys would require an increase of sample size for an independent HIV survey by 5.5%.

Approximately 6 000 respondents are expected to be involved in both TB and HIV components of the survey (1/4 of TB-component respondents and ½ of HIV-component participants).

For analyses, three sets of weights will need to be developed: TB only, another for HIV component (only), and a third for analysis of both TB-HIV.
